# Integrating SARS-CoV-2-specific interferon-γ release assay testing in the evaluation of patients hospitalized with COVID-19

**DOI:** 10.1128/spectrum.02419-23

**Published:** 2023-10-19

**Authors:** Mar Masiá, Alba de la Rica, Marta Fernández-González, José Alberto García, Sergio Padilla, Javier García-Abellán, Ángela Botella, Paula Mascarell, Félix Gutiérrez

**Affiliations:** 1 Infectious Diseases Unit, Hospital General Universitario de Elche, Alicant, Spain; 2 CIBER de Enfermedades Infecciosas, Instituto de Salud Carlos III, Madrid, Spain; 3 Clinical Medicine Department, Universidad Miguel Hernández, San Juan de Alicante, Spain; 4 Microbiology Service, Hospital General Universitario de Elche, Alicant, Spain; Quest Diagnostics Nichols Institute, Chantilly, Virginia, USA

**Keywords:** interferon-gamma release, IGRA, T cell immunity, COVID-19, SARS-CoV-2, mitogen, Omicron, outcomes, mortality, Delta, performance, accuracy

## Abstract

T cell-mediated immunity is crucial for protection against severe COVID-19. The performance of SARS-CoV-2-specific IFN-γ release assays (IGRAs) to measure T cell responses during severe acute infection is unknown. We conducted a prospective, longitudinal analysis of patients hospitalized for confirmed COVID-19. A standardized SARS-CoV-2-specific quantitative IGRA was measured on admission. Follow-up lasted 90 days. Two hundred forty-eight patients were included; 181 (73%) were vaccinated, 142 (57.3%) were infected with the Omicron variant, and 106 (42.7%) were infected with the Delta variant. The SARS-CoV-2-IGRA result was positive (>200 mIU/mL) in 125 (50.5%) patients, with 44 (35.2%) showing concomitant mitogen-induced IFN-γ response. Compared with patients with negative and borderline results, those with positive SARS-CoV-2 IGRA were younger and showed lower frequency of co-existing comorbidities, lower median Charlson comorbidity index, lower frequency of World Health Organization (WHO) severity score > 4, higher peripheral arterial oxygen saturation to inspired fraction of oxygen (SpO2/FiO2) ratio on admission, higher S-IgG and N-IgG antibody responses, and lower mortality at 28/60/90 days. These findings were consistent for both the Omicron and Delta variants. Similar patterns to those described above were observed in the subset of patients with positive SARS-CoV-2-IGRA and indeterminate mitogen-induced responses, as well as in those exhibiting both positive SARS-CoV-2-specific and mitogen responses. Twenty-eight (11.3%) patients had borderline (100–200 mIU/mL) SARS-CoV-2-IGRA results and shared several similarities with patients with negative SARS-CoV-2 IGRA. In an adjusted Cox model, a negative SARS-CoV-2-IGRA result was found to be an independent predictor of mortality. Using a receiver operating characteristics curve analysis, we found that an IFN-γ value of 150 mIU/mL was identified as the optimal cut-off point for predicting mortality, with 79% [95% confidence interval (CI) 61–93] sensitivity, 61% (95% CI 55–68) specificity, 18% (95% CI 15–22) positive predictive value, 96% (95% CI 93–98) negative predictive value, and an area under the curve (AUC) of 70%. The assay’s performance remained consistent regardless of mitogen results or the presence of the Delta or Omicron variants.

**IMPORTANCE:**

The cellular immune response is essential in the protection against severe disease in patients with established SARS-CoV-2 infection. The novelty of this study lies in the evaluation of the overall performance of a standardized assay to measure cellular immune response, the SARS-CoV-2-specific interferon-γ release assay (IGRA), in hospitalized patients with severe COVID-19. The SARS-CoV-2 IGRA was shown to accurately classify patients based on disease severity and prognosis, and the study revealed that test performance was not affected by the SARS-CoV-2 variant or control tube results. We identified an assay cut-off point with a high negative predictive value against mortality. The SARS-CoV-2 IGRA in patients hospitalized for COVID-19 may be a useful tool to assess cellular immunity and adopt targeted therapeutic and preventive measures.

## INTRODUCTION

The adaptive immune response is essential for the control and clearance of SARS-CoV-2 in patients with COVID-19 (1). T cell responses can be detected very early after SARS-CoV-2 infection, before the emergence of antibodies (2, 3), and provide long-lasting immunity against COVID-19 (4). Infection with SARS-CoV-2 elicits the activation of CD4+and CD8+ cytolytic T lymphocytes, both of which induce the production of interferon-gamma (IFN-γ). Additionally, CD4 +T cells aid in the proliferation and antibody secretion of B lymphocytes (5, 6). Thus, cellular immunity emerges as a critical axis in the immune response against SARS-CoV-2, exerting direct antiviral effects and regulating both the humoral and innate immune responses (6). Emerging data suggest a prominent role for T cells in protection against severe disease. Mild disease is characterized by an early T cell response (2, 7, 8), while insufficient or exhausted T cell responses are associated with severe COVID-19, even in vaccinated patients (9 – 11). Assessment of the magnitude and determinants of T cell responses in patients with acute severe COVID-19 may help adopting targeted preventive and therapeutic measures.

Measuring the SARS-CoV-2-specific T cell immunity is challenging for routine large-scale testing. The IFN-γ release assay (IGRA) is a simple and easy-to-perform test to evaluate the cellular immune response. While IGRAs are commonly used for assessing cellular immunity in mycobacterial infections (12), specific assays have emerged to evaluate T cell responses to SARS-CoV-2 by measuring IFN-γ production (13). The SARS-CoV-2 IGRA has been shown to accurately distinguish between convalescent and healthy uninfected individuals (14 – 16), identify subjects with symptomatic and asymptomatic infection after seroconversion (7), and estimate the immunity after vaccination in convalescent and uninfected persons (17 – 19). However, the performance of the SARS-CoV-2 IGRA in patients with acute infection needing hospital admission is unknown. Understanding the SARS-CoV-2 IFN-γ responses in patients with severe COVID-19 in an era when the majority of the population is vaccinated is essential for accurately interpreting test results and tailoring patient management accordingly.

We assessed the performance of SARS-CoV-2-specific IFN-γ release assay testing in patients hospitalized with COVID-19.

## MATERIALS AND METHODS

A prospective study was conducted on hospitalized COVID-19 patients between July 22, 2021, and April 23, 2022. Patients were selected based on microbiologically confirmed SARS-CoV-2 infection, abnormal chest x-ray findings, and/or severity criteria, including oxygen saturation < 93% and CURB-65 ≥ 2.

Clinical data and blood samples were collected according to a predefined protocol approved by the COVID-19 Institutional Committee (20, 21). The protocol included the standardized collection of clinical variables, nasopharyngeal samples (NPS) for SARS-CoV-2, and serial blood samples obtained on admission and every 48 h during hospital stay for routine blood tests and biomarker measurement, including levels of interleukin-6 (IL-6), ferritin, C-reactive protein (CRP), D-dimer, and lymphocyte counts and their subsets (AQUIOS CL Flow Cytometer, Beckman Coulter, USA). QuantiFERON-TB Gold Plus (Qiagen, Hilden, Germany) was performed on admission for screening for latent tuberculosis infection following the manufacturer’s instructions. Serum samples for the measurement of the levels of antibodies to SARS-CoV-2 were frozen at −80°C.

Patients received antiviral and/or immunomodulatory therapy containing dexamethasone ± remdesivir ± tocilizumab ± baricitinib according to pre-specified criteria (21, 22). All patients received antithrombotic prophylaxis with enoxaparin.

The study was approved by the Institutional Ethical Committee as part of the COVID-19 Elx/Spain project with Ethical Committee reference number PI 105/2021. Written informed consent for participation was not required for this study in accordance with the national legislation and the institutional requirements.

### Quantitative determination of IFN-γ release by SARS-CoV-2-specific T cells

Whole blood from lithium heparin tube was used for IGRA incubation within 4 h. SARS-CoV-2 cellular response was measured on the first day of admission using a specific quantitative IFN-γ release assay according to the manufacturer’s instructions (Quan-T-Cell SARS-CoV-2, Euroimmun, Germany). Details are provided elsewhere (9, 17). Briefly, lithium-heparinized blood from each patient was incubated for 21 h at 37°C in the three tubes supplied: blank tube (unstimulated) for the individual IFN-γ background and mitogen tube for non-specific IFN-γ secretion as controls and stimulation tube coated with antigens of the SARS-CoV-2 spike protein for specific IFN-γ secretion. The IFN-γ concentration released in the plasma fraction obtained after centrifugation of the three tubes was then measured by ELISA (Quant-T-Cell ELISA, Euroimmun, Germany) with an automated instrument (Dynex DS2 ELISA system). For non-specific IFN-γ release (mitogen-SARS-CoV-2 IGRA), a positive result was defined according to the manufacturer’s instructions as IFN-γ(mitogen) – IFN-γ(blank) ≥ 400 mUI/mL. For specific T cell response (SARS-CoV-2 IGRA), results were interpreted as follows: IFN-γ(SARS-CoV-2) – IFN-γ(blank) < 100 mIU/mL was considered negative, 100–200 borderline, and >200 positive. The performance of the specific SARS-CoV-2 IFN-γ response was assessed in both patients with and without a non-specific IFN-γ response. The upper limit of quantification achieved was 2500 mIU/mL.

### IgG against SARS-CoV-2 trimeric spike and internal nucleocapsid proteins

IgG levels against TrimericS-IgG on day 1 were quantified using a LIAISON SARS-CoV-2 TrimericS IgG Kit (DiaSorin, Italy) on a LIAISON XL analyzer. Criteria are as follows: ≥33.8 binding antibody units (BAU)/mL was positive (quantitative). N-IgG levels against internal N protein were measured using SARS-CoV-2 IgG assay (Abbott Laboratories, USA) on an Alinity i system. Interpretation is as follows: relative light unit (RLU)/calibrator RLU ratio < 1.40 = negative, ≥1.40 = positive.

### Characterization of SARS-CoV-2 variants

Confirmed SARS-CoV-2 PCR-positive NPS (Cobas 6800 System, Roche, Switzerland) were characterized either by sequencing or by multiplex PCR according to availability of resources. Genome sequencing was performed following the ARTIC amplicon sequencing protocol for MinION version V3 as previously described (23). Some NPS were characterized by a multiplex PCR (Novaplex SARSCoV2 Variants-VII Assay, Seegene, Korea), which targeted specifically the RdRP gene and E484A HV69/70del and N501Y mutations, following the manufacturer’s protocol, in a CFX96 real-time thermocycler (Bio-Rad, USA). The methodological details of the multiple PCR are described in the supplemental section (Suppl_Methods).

### Statistical analyses

Mann-Whitney-Wilcoxon or Student’s *t*-tests were used for group comparison in continuous variables, according to the result of Shapiro Wilk’s contrast of normality. For categorical variables, comparisons were performed using the *χ* ^2^ and Fisher’s exact tests of 2 categories and dichotomous variables, respectively. The correlation between the IFN-γ responses and the levels of antibodies and biomarkers was assessed with Pearson’s coefficient. We used multivariable adjusted Cox proportional hazard regression models to assess the association of the SARS-CoV-2 IGRA results with mortality incorporating covariates of interest. Receiver operating characteristics curves were used to select the best cut-off for IFN-γ concentration to predict mortality. Statistical analyses were performed with R version 4.0.3 software (R Foundation for Statistical Computing).

## RESULTS

### Study population

During the study period, 248 patients admitted to hospital met the criteria for inclusion in the study. The SARS-CoV-2 variant was Omicron in 142 (57.3%) patients and Delta in 106 (42.7%); in 155 (62.5%), it was confirmed by genome sequencing, in 74 (29.8%) by multiplex PCR, and in 19 (7.7%) cases with high real-time reverse-transcription PCR (RT-PCR) cycle threshold (Ct) values (≥35), it was assumed based on the prevalent variant circulating in our Department of Health at the time of infection. A total of 172 (69.4%) had a positive TrimericS-IgG antibody response. Patients’ characteristics on admission are shown in Table 1.

**TABLE 1:** Characteristics of persons admitted to the hospital with COVID-19 according to the presence of SARS-CoV-2 cellular response at hospital admission^*e*^

Characteristic	Negative SARS-CoV-2 IGRA (<100 mIU/mL)	Positive SARS-CoV-2 IGRA (>200 mIU/mL)	Borderline SARS-CoV-2 IGRA (100–200 mIU/mL)	All	*P* value
No, %	95 (38.3)	125 (50.4)	28 (11.3)	248	
Sex, male	54 (56.8)	64 (51.2)	16 (57.1)	134 (54.0)	0.672
Age, y	77 (58–85)	62 (49–79)	76 (63–83)	70 (52–83)	0.001
White, non-Hispanic	87 (91.6)	109 (87.2)	25 (89.3)	221 (89.1)	0.613
Charlson comorbidity index	4 (2–7)	3 (1–6)	4 (3–7)	4 (1–6)	0.078
Number of comorbidities^*a*^	2 (0–4)	1 (0–3)	2.5 (1 – 3)	2 (0–4)	0.056
Diabetes	27 (28.4)	27 (21.6)	6 (21.4)	60 (24.2)	0.506
Congestive heart failure	8 (8.4)	14 (11.2)	5 (17.9)	27 (10.9)	0.821
Coronary artery disease	15 (15.8)	12 (9.6)	3 (10.7)	30 (12.1)	0.411
Prior stroke	6 (6.3)	12 (9.6)	2 (7.1)	20 (8.1)	0.673
Peripheral arterial disease	5 (5.3)	9 (7.2)	1 (3.6)	15 (6.0)	0.803
Pulmonary disease	21 (22.1)	25 (20.0)	7 (25.0)	53 (21.4)	0.775
Chronic kidney disease	20 (21.1)	16 (12.8)	6 (21.4)	42 (16.9)	0.201
Malignant neoplasm	12 (12.6)	17 (13.6)	4 (14.3)	33 (13.3)	0.965
Immunosuppressive condition*^b^*	9 (9.5)	18 (14.4)	6 (21.4)	33 (13.3)	0.214
Prior COVID-19 infection	1 (1.1)	5 (4.0)	3 (10.7)	9 (3.6)	0.046
Vaccination status					
Unvaccinated	28 (29.5)	33 (26.4)	6 (21.4)	67 (27.0)	0.706
Vaccinated*^c^*	67 (70.5)	92 (73.6)	22 (78.6)	181 (73.0)	
Days since last vaccine dose	119 (68–186)	95 (59–175)	110 (81–175)	100 (67–178)	0.252
Clinical presentation					
Days from symptom onset to admission	5 (3–7)	6 (3–8)	5 (2–7)	5 (3–7)	0.632
SpO2/FiO2 ratio at admission	343 (283–354)	354 (343–462)	346 (294–417)	350 (309–457)	0.001
WHO severity score	4 (3.1–4.1)	4 (3.0–4.0)	4 (3.9–4.0)	4 (3.1–4.1)	0.386
WHO severity score > 4	12 (12.6)	4 (3.2)	2 (7.1)	18 (7.3)	0.022
X-Ray bilateral lung infiltrates	44 (46.3)	62 (49.6)	12 (42.9)	118 (47.6)	0.568
Microbiological data					
SARS-CoV-2 variant, Omicron	58 (61.1)	69 (55.2)	15 (53.6)	142 (57.3)	0.611
Lowest cycle threshold PCR	22 (17–26)	23 (19–28)	23 (19–29)	22 (18–29)	0.202
TrimericS-IgG, BAU/mL	143 (5–2535)	1090 (81–3650)	261 (6–2218)	581 (10–3278)	0.053
TrimericS-IgG, positive	56 (58.9)	99 (79.2)	17 (60.7)	172 (69.4)	0.003
TrimericS-IgG > 264 BAU/mL	45 (47.4)	82 (65.6)	14 (50.0)	141 (56.9)	0.019
N-IgG, positive	8 (8.4)	23 (18.4)	3 (10.7)	34 (13.7)	0.095
SARS-CoV-2 IGRA, mIU/mL	10 (0.5–43)	635 (337–1752)	163 (139–173)	206 (26–636)	<0.001
SARS-CoV-2 IGRA, positive	0 (0)	125 (100)	0 (0)	125 (50.4)	<0.001
Mitogen-SARS-CoV-2 IGRA^*d*^ positive	12 (12.6)	44 (35.2)	6 (21.4)	62 (25.0)	<0.001
Mitogen-QuantiferonTB, positive (≥0.50 IU/mL)	77/84 (91.7)	105/110 (95.5)	25/26 (96.2)	207/220 (94.1)	0.692
QuantiferonTB, positive	3 (3.5)	4 (3.6)	1 (3.7)	8 (3.6)	1.000
Antivirals/immunomodulators					
Remdesivir	79 (83.2)	98 (78.4)	24 (85.7)	201(81.0)	0.573
Monoclonal antibodies	4 (4.2)	2 (1.6)	0 (0)	6 (2.4)	0.399
Tocilizumab or baricitinib	59 (62.1)	85 (68.0)	19 (67.9)	163 (65.7)	0.656
Outcomes					
Hospital stay, days	5 (3.5–9)	5 (3–7)	7 (4–8)	5 (3–8)	0.171
ICU admission	7 (7.4)	3 (2.4)	2 (7.1)	12 (4.8)	0.147
28-d mortality	15 (15.8)	5 (4.0)	4 (14.3)	24 (9.7)	0.006
60-d mortality	19 (20.0)	10 (8.0)	5 (17.9)	34 (13.7)	0.025
90-d mortality	21 (22.1)	10 (8.0)	5 (17.9)	36 (14.5)	0.009

^*a*^ This category included the following underlying medical conditions: diabetes mellitus, chronic cardiac disease, chronic kidney disease, chronic liver disease, chronic neurologic disease, chronic pulmonary disease, malignancies, and immunosuppressive conditions.

^*b*^ This category included HIV, solid or bone marrow transplant, active hematologic malignancy, receiving immunosuppression, or active chemotherapy.

^*c*^ Of 181 vaccinated patients, 103 (57%) were fully vaccinated and 78 (43%) had received a booster dose.

^*d*^ A positive result was defined according to the manufacturer as follows: IFN-γ(mitogen) – IFN-γ(blank) ≥ 400 mIU/mL. Data are presented as no. (%) unless otherwise indicated. IGRA, SARS-CoV-2 interferon gamma release assay; SpO2/FiO2 ratio, oxygen saturation to fraction of inspired oxygen ratio; TrimericS-IgG, immunoglobulin G antibody serum levels against the trimeric spike protein; BAU, binding antibody units; ICU, intensive care unit.

^*e*^ Continuous variables are expressed as median (interquartile range). Categorical variables are expressed as number (percentage).

### Performance of the SARS-CoV-2 IFN-γ release assay

The SARS-CoV-2 IGRA result was positive (>200 mIU/mL) in 125 (50.5%) patients. Patients with positive SARS-CoV-2 IGRA results showed a differential clinical profile (Table 1). Compared with patients with either negative or borderline results, those with positive SARS-CoV-2 IGRA were younger, showed lower frequency of co-existing comorbidities, lower median Charlson comorbidity index, lower frequency of WHO severity score > 4, and higher peripheral arterial oxygen saturation to inspired fraction of oxygen (SpO2/FiO2) ratio on admission (Table S5).

The TrimericS-IgG and N-IgG positive responses were more frequent among SARS-CoV-2 IGRA-positive patients, with no differences according to SARS-CoV-2 Ct values. There was a mild correlation between the TrimericS-IgG and SARS-CoV-2 IGRA values, with a *ρ* = 0.17 (*P* < 0.001) (Fig. S1). This correlation did not achieve statistical significance in the group of patients who died (*ρ* = 0.017; *P* = 0.935). The QuantiFERON-TB was positive in 3.6% of patients, indeterminate in 5.9% of patients (of them, 0.4% had a positive SARS-CoV-2 IGRA mitogen result and 2.2% had a positive value for the specific SARS-CoV-2-IGRA), and the QuantiFERON-TB mitogen test was positive in 94.1% of patients tested, with no differences according to SARS-CoV-2 IGRA results. Patient outcomes differed by SARS-CoV-2 IGRA status, with lower mortality at 28, 60 and 90 days after hospital admission among SARS-CoV-2 IGRA-positive patients and a trend to lower admission to the intensive care unit (ICU) (*P* = 0.083) (Table 1).

Table 2 shows the results of the SARS-CoV-2 IGRA according to lymphocyte counts and biomarker levels. Positive SARS-CoV-2 IGRA was associated with a higher number of total peripheral lymphocyte, B cell, T cell, and CD4+ T cell counts and a trend to a lower number of natural killer (NK) cells. Less than one-third of patients with T cells of less than 200/µL showed positive SARS-CoV-2 IGRA results, and 91.4% of them showed a negative result for the SARS-CoV-2 IGRA mitogen. Of the biomarkers measured, the positive SARS-CoV-2 IGRA was associated with higher IL-6 levels and a trend to lower ferritin levels on admission. There was a mild, negative correlation of the IFN-γ response with C-reactive protein and D-dimer (*ρ* = −0.11, *P* < 0.05 in both cases; Fig. S1)

**TABLE 2:** Lymphocyte counts and biomarker levels on admission^*a*^

Characteristic	Negative SARS-CoV-2 IGRA (<100 mIU/mL)	Positive SARS-CoV-2 IGRA (>200 mIU/mL)	Borderline SARS-CoV-2 IGRA (100–200 mIU/mL)	All	*P* value
Peripheral lymphocyte count, cells/μL	606 (419–951)	795 (498–1,070)	763 (544–957)	694 (461–1,013)	0.048
Peripheral lymphocyte count, categories, cells/μL					
<100	1 (1.1)	1 (0.8)	0 (0.0)	2 (0.8)	
100–199	2 (2.1)	4 (3.3)	0 (0.0)	6 (2.5)	
200–500	35 (37.2)	27 (22.0)	5 (19.2)	67 (27.6)	0.161
>500	56 (59.6)	91 (74.0)	21 (80.8)	168 (69.1)	
T cell count, cells/μL	367 (224–566)	468 (314–739)	503 (406–598)	435 (281–660)	0.013
T cell count, category, cells/μL					
<100	3 (3.2)	2 (1.6)	0 (0.0)	5 (2.1)	
100–199	19 (20.2)	9 (7.3)	2 (7.7)	30 (12.3)	
200–500	40 (42.6)	54 (43.9)	11 (42.3)	105 (43.2)	0.081
>500	32 (34.0)	58 (47.2)	13 (50.0)	103 (42.4)	
CD4+ T cell count, cells/μL	193 (110–327)	277 (153–414)	342 (272–373)	240 (146–376)	0.003
CD4+ T cell count, category, cells/μL					
<100	19 (20.2)	16 (13.0)	2 (7.7)	37 (15.2)	
100–199	29 (30.9)	30 (24.4)	2 (7.7)	61 (25.1)	
200–500	34 (36.2)	54 (43.9)	20 (76.9)	108 (44.4)	0.012
>500	12 (12.8)	23 (18.7)	2 (7.7)	37 (15.2	
CD4+/CD8 lymphocyte ratio	1.4 (0.9–2.3)	1.6 (1.0–2.4)	2.0 (1.3–2.8)	1.6 (1.0–2.4)	0.102
B cell count, cells/μL	54 (23–89)	65 (38–126)	58 (36–103)	60 (28–113)	0.033
NK lymphocytes/μL	23 (14–34)	19 (12–29)	21 (14–30)	20 (13–31)	0.076
Interleukin-6, pg/mL	29 (6–131)	41 (16–134)	80 (42–280)	42 (12–139)	0.026
C-Reactive protein, mg/L	49 (23–103)	53 (24–88)	47 (29–104)	50 (24–97)	0.930
D-Dimer, μg/mL	0.8 (0.4–2.3)	0.7 (0.4–1.1)	0.7 (0.5–1)	0.7 (0.4–1.4)	0.316
Ferritin, ng/mL	248 (104–519)	199 (100–441)	431 (167–609)	231 (108–503)	0.075

^*a*^ Continuous variables are expressed as median (interquartile range). Categorical variables are expressed as number (percentage).

Performance of the SARS-CoV-2 IGRA according to patients’ demographic and clinical data is shown in Table S1. Results were consistent with those described above. There were no differences in the SARS-CoV-2 IGRA results by SARS-CoV-2 variant. Patients who died at all time points showed higher frequency of negative SARS-CoV-2 IGRA, and the same trend was observed among patients admitted to the ICU.

A total of 181 (73%) patients were vaccinated. There were no significant differences in the positivity of SARS-CoV-2 IGRA according to vaccination status or days since the last vaccine dose. Vaccinated patients were older, had a higher Charlson comorbidity index and higher burden of comorbidities, and showed a trend to higher frequency of positive mitogen (Table S2).

In the analysis by SARS-CoV-2 variant, patients infected with Delta variants were significantly younger and showed lower comorbidity burden, lower frequency of vaccination, and higher number of days since symptom initiation (Table S3). When positive and negative SARS-CoV-2 IGRA results were compared in patients with Omicron and Delta variants separately, the patterns were similar to those observed in the entire cohort (Table 3). Among patients infected with the Delta variant, individuals with a negative SARS-CoV-2 IGRA result showed a 28-day mortality rate that was twice as high as those with a positive result (13.5% vs. 5.4%), but the disparity was not statistically significant.

**TABLE 3:** Characteristics of persons admitted to the hospital with the Omicron or Delta variant, according to the presence of SARS-CoV-2 cellular response at hospital admission^*d*^

	Delta			Omicron		
Characteristics	Negative SARS-CoV-2 IGRA	Positive SARS-CoV-2 IGRA	*P* Value	Negative SARS-CoV-2 IGRA	Positive SARS-CoV-2 IGRA	*P* Value
*n* (%)	50 (47.2)	56 (52.8)		73 (51.4)	69 (48.6)	
Sex, male	37 (74.0)	24 (42.9)	0.002	33 (45.2)	40 (58.0)	0.135
Age, y	67 (55–80)	56 (49–72)	0.006	81 (60–86)	73 (51–83)	0.014
Charlson comorbidity index	3 (1–7)	2 (1–3)	0.036	5 (3–7)	5 (1–7)	0.622
Number of comorbidities^*a*^	1 (0–4)	0.5 (0–2)	0.011	2 (1–4)	2 (0–4)	0.714
Diabetes	16 (32.0)	8 (14.3)	0.037	17 (23.3)	19 (27.5)	0.570
Congestive heart failure	3 (6.0)	4 (7.1)	1.000	10 (13.7)	10 (14.5)	1.000
Coronary artery disease	6 (12.0)	3 (5.4)	0.301	12 (16.4)	9 (13.0)	0.640
Prior stroke	3 (6.0)	2 (3.6)	0.665	5 (6.8)	10 (14.5)	0.176
Peripheral arterial disease	3 (6.0)	1 (1.8)	0.341	3 (4.1)	8 (11.6)	0.122
Pulmonary disease	7 (14.0)	9 (16.1)	0.793	21 (28.8)	16 (23.2)	0.567
Chronic kidney disease	10 (20.0)	4 (7.1)	0.082	16 (21.9)	12 (17.4)	0.533
Malignant neoplasm	5 (10.0)	5 (8.9)	1.000	11 (15.1)	12 (17.4)	0.821
Immunosuppressive condition^*b*^	3 (6.0)	5 (8.9)	0.720	2 (1–4)	(0–4)	0.714
Prior COVID-19 infection	3 (6.0)	0	3 (6.0)	1 (1.4)	5 (7.2)	0.109
Vaccination status						
Days since last vaccine dose	129 (80–180)	128 (62–180)	0.697	104 (73–188)	84 (56–157)	0.084
Unvaccinated	12 (24.0)	16 (28.6)	0.662	22 (30.1)	17 (24.6)	0.573
Fully vaccinated	38 (76.0)	40 (71.4)		51 (69.9)	52 (75.4)	
Clinical presentation						
Days from symptom onset admission	6 (5–7)	6 (4–7)	0.923	4 (2–7)	4 (2–8)	0.363
SpO2/FiO2 ratio at admission	346 (301–450)	350 (327–462)	0.119	343 (274–408)	354 (343–462)	0.003
WHO severity score > 4	8 (16.0)	2 (3.6)	0.044	6 (8.2)	2 (2.9)	0.276
X-Ray bilateral lung infiltrates	25 (50.0)	33 (58.9)	0.110	31 (42.5)	29 (42.0)	0.926
Microbiological data						
Lowest cycle threshold PCR	23 (18–30)	25 (21–29)	0.565	20 (17–27)	21 (18–28)	0.159
TrimericS-IgG, BAU/mL	196 (10–5495)	515 (85–4140)	0.521	143 (5–1800)	1470 (57–2920)	0.006
TrimericS-IgG, positive	32 (64.0)	46 (82.1)	0.047	41 (56.2)	53 (76.8)	0.013
TrimericS-IgG > 264 BAU/mL	24 (48.0)	34 (60.7)	0.242	35 (47.9)	48 (69.6)	0.011
N-IgG, positive	6 (12.0)	11 (19.6)	0.305	5 (6.8)	12 (17.4)	0.070
SARS-CoV-2 IGRA, mIU/mL	45 (12–113)	863 (343–1838)	<0.001	11 (0.5–63)	575 (337–1400)	<0.001
Mitogen-SARS-CoV-2 IGRA,^*c*^ positive	3 (6.0)	20 (35.7)	<0.001	15 (20.5)	24 (34.8)	0.063
Laboratory values						
Interleukin-6, pg/mL	60 (12–147)	42 (24–116)	0.985	30 (6–140)	36 (11–143)	0.668
C-Reactive protein, mg/L	64 (29–99)	60 (32–98)	0.927	39 (19–104)	40 (21–75)	0.628
D-Dimer, μg/mL	0.7 (0.5–1.1)	0.6 (0.4–1.0)	0.169	0.9 (0.4–2.2)	0.8 (0.5–1.4)	0.547
Ferritin, ng/mL	418 (229–598)	293 (154–535)	0.262	173 (91–529)	164 (80–321)	0.263
Peripheral lymphocyte count, cells/μL	644 (424–940)	823 (519–1212)	0.056	633 (457–962)	708 (492–978)	0.470
B cell count, cells/μL	57 (29–109)	64 (36–110)	0.679	49 (19–85)	69 (40–133)	0.005
T cell count, cells/μL	370 (254–567)	562 (329–830)	0.024	440 (261–574)	425 (314–709)	0.444
CD4+ T cell count, cells/μL	206 (99–357)	326 (157–473)	0.003	231 (149–369)	214 (151–371)	0.861
CD4+/CD8 lymphocyte ratio	1.1 (0.7–2.2)	1.6 (1.0–2.4)	0.143	1.9 (1.1–2.4)	1.8 (1.2–2.3)	0.788
Antivirals/immunomodulators						
Remdesivir	43 (86.0)	43 (76.8)	0.320	60 (82.2)	55 (79.7)	0.831
Monoclonal antibodies	0	1 (1.8)	1.000	4 (5.5)	1 (1.4)	0.230
Tocilizumab or baricitinib	40 (80.0)	44 (78.6)	1.0	38 (52.1)	41 (59.4)	0.402
Outcomes						
Hospital stay	5 (3–7)	4 (3–7)	0.594	6 (4–10)	5 (3–7)	0.115
28-d mortality	5 (13.5)	3 (5.4)	0.370	13 (17.8)	2 (2.9)	0.005
60-d mortality	6 (16.2)	4 (7.1)	0.358	17 (23.3)	6 (8.7)	0.023
90-d mortality	6 (16.2)	4 (7.1)	0.358	19 (26.0)	6 (8.7)	0.008

^*a*^ This category included the following underlying medical conditions: diabetes mellitus, chronic cardiac disease, chronic kidney disease, chronic liver disease, chronic neurologic disease, chronic pulmonary disease, malignancies, and immunosuppressive conditions.

^*b*^ This category included HIV, solid or bone marrow transplant, active hematologic malignancy, receiving immunosuppression, or active chemotherapy.

^*c*^ A positive result was defined according to the manufacturer as follows: IFN-γ(mitogen) – IFN-γ(blank) ≥ 400 mIU/mL. Data are presented as no. (%) unless otherwise indicated. IGRA, SARS-CoV-2 interferon gamma release assay; SpO2/FiO2 ratio, oxygen saturation to fraction of inspired oxygen ratio; TrimericS-IgG, immunoglobulin G antibody serum levels against the trimeric spike protein; BAU, binding antibody units; ICU, intensive care unit.

^*d*^ Continuous variables are expressed as median (interquartile range). Categorical variables are expressed as number (percentage).

### Borderline SARS-CoV-2 IFN-γ release assay results

A total of 28 (11.3%) patients showed borderline (100–200 mIU/mL) SARS-CoV-2 IGRA results. They shared several similarities with SARS-CoV-2 IGRA-negative patients in demographic characteristics, comorbidity burden, and TrimericS-IgG and N-IgG antibody responses, although they exhibited higher T cell and CD4+ T cell counts, higher ferritin and IL-6 levels, with the latter showing the highest numerical values observed among the groups, and higher frequency of prior COVID-19 (Table 1). The frequency rates of ICU admission and mortality at 28, 60, and 90 days were also similar to those of SARS-CoV-2 IGRA-negative patients.

### Mitogen (non-specific) IFN-γ responses

Of 125 SARS-CoV-2 IGRA-positive patients, 44 (35.2%) had concomitant positive mitogen-induced IFN-γ response. These patients showed a lower WHO severity score, higher initial SpO2/FiO2, higher frequency of vaccination, and higher number of total, T, and CD4+ T lymphocytes than those with only SARS-CoV-2-specific IFN-γ responses (Table 4). The characteristics of the 81 (64.8%) SARS-CoV-2 IGRA-positive patients with indeterminate mitogen-induced response were compared with those of patients with negative SARS-CoV-2 IGRA. They exhibited numerous similarities to the overall group of SARS-CoV-2 IGRA-positive patients in terms of demographics, comorbidities, severity criteria upon admission, and TrimericS-IgG and N-IgG antibody responses. They also showed lower mortality at all time points (Table 4). Comparison of patients with positive SARS-CoV-2 IGRA and either specific or non-specific IFN-γ responses, with patients with negative SARS-CoV-2 IGRA, showed similar results (Table 4). A second sample for SARS-CoV2 IGRA was obtained from 53 patients during follow-up [median Q1–Q3, 54 (48–57) days]. Of 41 (77.3%) patients with indeterminate initial mitogen response, 36 (87.8%) switched to positive response, and of 23 with initial negative specific response, 13 (57%) changed to positive response.

**TABLE 4:** Characteristics of persons hospitalized with COVID-19 with SARS-CoV-2 IGRA-positive and concomitant positive mitogen-SARS-CoV-2 IGRA^*g*^

Characteristic	S-IGRA (+) &^*f*^ M-IGRA (+)	S-IGRA (+) & M-IGRA (−)	*P* ^*a*^	S-IGRA (−)	*P* ^*b*^	*P* ^*c*^
*n (%)*	44 (31.7)	81 (46.0)		95 (68.3)		
Sex, male	20 (45.5)	44 (54.3)	0.356	54 (56.8)	0.273	0.763
Age	62 (48–79)	62 (50–79)	0.924	77 (58–85)	0.011	0.001
Charlson comorbidity index	2 (1–6)	2 (1–6)	0.697	4 (2–7)	0.243	0.032
Number of comorbidities^*d*^	1 (0–4)	1 (0–3)	0.910	2 (0–4)	0.186	0.111
Diabetes	11 (25.0)	16 (19.8)	0.503	27 (28.4)	0.838	0.219
Congestive heart failure	5 (11.4)	9 (11.1)	1.000	8 (8.4)	0.549	0.614
Coronary artery disease	5 (11.4)	7 (8.6)	0.752	15 (15.8)	0.608	0.176
Prior stroke	6 (13.6)	6 (7.4)	0.342	6 (6.3)	0.195	0.775
Peripheral arterial disease	2 (4.5)	7 (8.6)	0.491	5 (5.3)	1.000	0.389
Pulmonary disease	7 (15.9)	18 (22.2)	0.498	21 (22.1)	0.498	1.000
Chronic kidney disease	4 (9.1)	12 (14.8)	0.416	20 (21.1)	0.096	0.330
Malignant neoplasm	5 (11.4)	12 (14.8)	0.786	12 (12.6)	1.000	0.826
Immunosuppressive condition^*e*^	6 (13.6)	12 (14.8)	0.550	9 (9.5)	0.558	0.352
Prior COVID-19 infection	1 (2.3)	4 (4.9)	0.656	1 (1.1)	0.534	0.182
Vaccination status						
Days since last vaccine dose	83 (54–157)	99 (64–179)	0.317	119 (68–186)	0.149	0.496
Unvaccinated	6 (13.6)	27 (33.3)	0.047	28 (29.5)	0.056	0.626
Clinical presentation						
Days from symptom onset admission	5 (2–7)	6 (3–8)	0.195	5 (3–7)	0.545	0.174
SpO2/FiO2 ratio at admission	452 (350–467)	350 (323–457)	0.005	343 (275–413)	<0.001	0.036
WHO severity score > 4	1 (2.3)	3 (3.7)	0.038	12 (12.6)	0.001	0.323
X-Ray bilateral lung infiltrates	16 (36.4)	46 (56.8)	0.220	44 (46.3)	0.374	0.115
Microbiological data						
SARS-CoV-2 variant, Omicron	24 (54.5)	45 (55.6)	1.000	58 (61.1)	0.578	0.540
Lowest cycle threshold PCR	23 (18–26)	24 (19–30)	0.198	22 (17–26)	0.262	0.100
TrimericS-IgG, BAU/mL	1420 (334–4723)	608 (19–2800)	0.087	143 (4.8–2535)	0.008	0.152
TrimericS-IgG, positive	39 (88.6)	60 (74.1)	0.067	56 (58.9)	<0.001	0.039
TrimericS-IgG > 264 BAU/mL	33 (75.0)	49 (60.5)	0.007	45 (47.4)	0.003	0.096
N-IgG, positive	4 (9.1)	19 (23.5)	0.055	8 (8.4)	1.000	0.007
SARS-CoV-2 IGRA, mIU/mL	1269 (551–2500)	521 (310–1334)	<0.001	10 (0.5–43)	<0.001	<0.001
Laboratory values						
Interleukin-6, pg/mL	45 (16–177)	37 (16–106)	0.330	29 (6–131)	0.125	0.290
C-Reactive protein, mg/L	48 (22–74)	54 (26–97)	0.220	49 (23–103)	0.292	0.828
D-Dimer, μg/mL	0.5 (0.3–1.0)	0.8 (0.5–1.2)	0.012	0.8 (0.4–2.3)	0.014	0.660
Ferritin, ng/mL	175 (97–244)	279 (117–545)	0.009	248 (104–519)	0.023	0.598
Peripheral lymphocyte count, cells/μL	835 (655–1508)	712 (420–980)	0.008	606 (419–951)	<0.001	0.414
B cell count, cells/μL	77 (41–147)	64 (37–120)	0.296	54 (23–89)	0.010	0.052
T cell count, cells/μL	636 (429–1058)	405 (270–591)	0.001	367 (224–566)	<0.001	0.269
CD4+ T cell count, cells/μL	371 (192–624)	211 (145–359)	0.001	193 (110–327)	<0.001	0.206
CD4+/CD8 lymphocyte ratio	2.0 (1.3–2.5)	1.5 (1.0–2.2)	0.100	1.4 (0.9–2.3)	0.051	0.519
Antiviral/immunomodulator						
Remdesivir	35 (79.5)	63 (77.8)	0.664	79 (83.2)	0.639	0.444
Monoclonal antibodies	43 (97.7)	1 (1.2)	0.578	91 (95.8)	1.000	0.376
Tocilizumab or baricitinib	15 (34.1)	25 (30.9)	0.630	36 (37.9)	0.709	0.345
Outcomes						
28-d mortality	1 (2.3)	4 (4.9)	0.656	15 (15.8)	0.021	0.027
60-d mortality	4 (9.1)	6 (7.4)	0.740	19 (20.0)	0.142	0.018
90-d mortality	4 (9.1)	6 (7.4)	0.740	21 (22.1)	0.095	0.011

^*a*^ *P* for the comparison of positive mitogen and negative mitogen results among SARS-CoV-2 IGRA-(+) patients.

^*b*^ P for the comparison of IGRA(+) & mitogen(+) patients with IGRA(−) patients.

^*c*^ P for the comparison of IGRA(+) & mitogen(−) patients with IGRA(−) patients.

^*d*^ This category included the following underlying medical conditions: diabetes mellitus, chronic cardiac disease, chronic kidney disease, chronic liver disease, chronic neurologic disease, chronic pulmonary disease, malignancies, and immunosuppressive conditions.

^*e*^ This category included HIV, solid or bone marrow transplant, active hematologic malignancy, receiving immunosuppression, or active chemotherapy.

^*f*^ A positive result was defined according the manufacturer as follows: IFN-γ(mitogen) – IFN-γ(blank) ≥ 400 mUI/mL. Data are presented as no. (%) unless otherwise indicated. IGRA, interferon gamma release assay; S-IGRA, SARS-CoV-2 IGRA; M-IGRA, non-specific (mitogen) IGRA response; SpO2/FiO2 ratio, oxygen saturation-fraction of inspired oxygen ratio; TrimericS-IgG, immunoglobulin G antibody serum levels against the trimeric spike protein; BAU, binding antibody units; ICU, intensive care unit.

^*g*^ Continuous variables are expressed as median (interquartile range). Categorical variables are expressed as number (percentage).

### Accuracy of the SARS-CoV-2 IFN-γ release assay to predict patient outcomes

A total of 24 (9.7%) patients died within 28 days after hospital admission. Patients’ characteristics according to disease outcomes are shown in Table S4. Mortality was associated with older age, higher comorbidity burden, higher disease severity, lower frequency of therapy with remdesivir, and higher levels of all biomarkers. In a Cox model adjusted for age, comorbidity burden, WHO severity score, positive TrimericS-IgG, lymphocyte counts, and remdesivir therapy, the negative SARS-CoV-2 IGRA was an independent predictor of mortality. Additionally, the comorbidity burden and a WHO severity score > 4 were also identified as independent predictors of mortality (Table 5). The sensitivity rate of a negative SARS-CoV-2 IGRA result to predict mortality was 63% [95% confidence interval (CI) 57–69], specificity 54% (95% CI 48–60), positive predictive value (PPV) 16% (95% CI 11–21), and negative predictive value (NPV) 96% (95% CI 93–98). Using a receiver operating characteristics (ROC) curve, an IFN-γ cut-off value of 150 mIU/mL had a 79% (95% CI 61–93) sensitivity, 61% (95% CI 55–68) specificity, 18% (95% CI 15–22) PPV, 96% (95% CI 93–98) NPV, and area under the curve (AUC) of 70% to predict mortality (Fig. S2).

**TABLE 5:** Adjusted Cox model of predictors of 28-day mortality in persons admitted to the hospital with COVID-19^*a*^

Variable	Unadjusted HR (95% CI)	Unadjusted *P*	Adjusted HR (95% CI)	Adjusted *P*
Age >64 y	9.51 (2.24–40.43)	*P* = 0.002	1.99 (0.43–9.29)	0.381
Charlson comorbidity index	1.32 (1.18–1.48)	*P* < 0.001	1.42 (1.18–1.69)	<0.001
WHO severity score > 4	4.02 (1.50–10.78)	*P* = 0.006	3.34 (1.14–9.74)	0.028
Positive SARS-CoV-2 IGRA	0.24 (0.09–0.65)	*P* = 0.005	0.24 (0.08–0.75)	0.009
Borderline SARS-CoV-2 IGRA	0.88 (0.29–2.65)	*P* = 0.817	1.04 (0.33–3.28)	0.946
Positive TrimericS-IgG	4.02 (1.50–10.78)	*P* = 0.006	0.47 (0.18–1.22)	0.122
Peripheral lymphocyte count (log 10), cells/μL	0.45 (0.18–1.14)	*P* = 0.093	0.92 (0.35–2.38)	0.857
Remdesivir	0.36 (0.16–0.83)	*P* = 0.016	0.41 (0.17–0.97)	0.053

^*a*^ HR, hazard ratio; CI, confidence interval.

## DISCUSSION

Using a specific standardized SARS-CoV-2 IGRA, our study reveals a substantial proportion of negative results upon admission among patients hospitalized for COVID-19 during the vaccination era. Older age, greater comorbidity burden, increased disease severity, lower total and T lymphocyte counts, and absence of prior infection were associated with a higher frequency of negative SARS-CoV-2 IGRA results. However, vaccination status did not impact the likelihood of negative IGRA outcomes. Neither the mitogen results nor the presence of SARS-CoV-2 Omicron or Delta variant affected the performance of the test. Initial negative SARS-CoV-2 IFN-γ response was predictive of increased mortality.

To assess the role of T cells to provide protection and limit disease severity in SARS-CoV-2 infection, the magnitude and timing of the immune response should be analyzed in individuals with acute disease. The novelty of this study lies in the evaluation of the overall performance of a standardized SARS-CoV-2 IGRA in a non-selected cohort of vaccinated and unvaccinated patients with severe COVID-19 upon hospital admission, potentially enabling tailored patient management based on the results. The commercial SARS-CoV-2 IGRA utilized in this study has previously demonstrated simplicity and reliability in quantifying T cell response (17), offering the advantage of easy implementation and potential applicability to a large number of samples. Our findings revealed a high frequency of negative SARS-CoV-2 IGRA results in hospitalized COVID-19 patients, which significantly influenced patient outcomes. Negative SARS-CoV-2 IGRA was frequent among patients with older age and higher comorbidity burden, consistent with the reduced T cell responses reported in both scenarios (24, 25). Aging has been associated with waning T cell immune competence, at the level of both the T cell population and its functionality (23). Unexpectedly, there were no differences in the SARS-CoV-2 IGRA results by vaccination status, with half of the vaccinated patients showing negative results. Vaccinated patients were older and had a higher burden of comorbidity. While no specific comorbidity was associated with negative SARS-CoV-2 IGRA results, the accumulation of comorbidities predicted lower IFN-γ release. Our results are in agreement with the reduced and gradually lower CD4+ spike-specific T cell responses after vaccination reported with higher prevalence of comorbidities and older age (26). Current vaccination strategies may therefore be insufficient to induce a protective cellular immune response to avoid severe COVID-19 in specific groups of patients, in whom alternative preventive measures such as vaccines designed to induce T cell immunity (27) in addition to anticipated therapy may be considered. We did not find differences in the proportion of negative IGRA results in the 33 immunosuppressed patients included in our cohort, to which different factors including the sample size and the definition of immunosuppression might have accounted for.

Negative SARS-CoV-2 IGRA results were associated with increased disease severity, higher peripheral lymphocytopenia, and decreased T cell and CD4+ T cell counts. Lymphocytopenia is a hallmark of severe disease in patients with COVID-19 (28), primarily involving T cells, both CD4+ and CD8+ lymphocytes (6, 23, 29, 30), which may account for the lower IFN-γ levels detected (23, 30). Reduced peripheral lymphocyte counts and, less frequently, decreased CD4+ counts have been linked to false-negative IGRA results in patients with tuberculosis (31) and indeterminate *QuantiFERON-TB Gold Plus* assay results in COVID-19 patients (32). While false-negative SARS-CoV-2 IGRA results have not been reported in severe COVID-19 patients, a shift from negative to positive IFN-γ response was observed as the infection resolved and lymphocyte count recovered in a subset of patients with follow-up samples, similar to findings with the *QuantiFERON-TB Gold Plus* test (33). Importantly, these initial negative results were predictive of poorer outcomes. Patients with negative SARS-CoV-2 IGRA also showed lower B cell counts, reflecting a generalized blunted lymphocyte response associated with acute infection. However, 60% of them had detectable S-IgG antibody levels, which supports the greater impact of acute SARS-CoV-2 infection on T cell than B cell responses. Actually, changes in B cells and NK cells in patients with severe disease have been reported to be much less pronounced than those observed with T cells, as well as considerable inter-individual variation in B cell numbers despite lymphopenia (23, 29, 30, 34).

SARS-CoV-2 IGRA results were found to be associated with patient outcomes, as we have seen in a preliminary analysis conducted in the vaccinated patient group (9). SARS-CoV-2 IGRA-negative persons exhibited higher mortality rates, irrespective of age, comorbidity burden, initial severity, and the antibody response. Interferon-γ plays a critical role in early macrophage activation, leading to the phagocytosis and clearance of SARS-CoV-2 (35). Accordingly, an inadequate early T cell response in patients with established infection contributes to SARS-CoV-2 persistence and disease progression (8). We identified a threshold for the protective effect of the SARS-CoV-2 IGRA. Although the area under the curve was suboptimal, an IFN-γ result above 150 mIU/mL showed a high negative predictive value against mortality. The SARS-CoV-2 IGRA results at hospital admission provide clinicians with valuable information regarding patients’ prognosis and may assist in guiding decisions about the timing and intensity of anti-COVID therapy or duration of hospitalization.

Despite the younger age and lower comorbidity burden of patients infected with the Delta variant, the performance of SARS-CoV-2 IGRA was similar to that observed in patients infected with the Omicron variant and the entire cohort. In patients infected with the Delta variant, the differences in mortality rates between positive and negative SARS-CoV-2 IGRA results did not reach statistical significance. However, the proportion of deaths in the SARS-CoV-2 IGRA-negative group was twice as high as that in the positive group, and the number of deaths was lower compared with that of the Omicron variant. While results regarding the performance of SARS-CoV-2 IGRA cannot be extrapolated to other variants, our data align with the low impact of SARS-CoV-2 variants on T cell responses in infected or vaccinated individuals (36).

In our cohort, there was a relatively low percentage of individuals with a history of confirmed COVID-19. However, this percentage increased fourfold when considering patients who displayed an immune response indicative of prior infection (i.e., positive N-IgG titers). This finding suggests a high proportion of asymptomatic infections among the patients. Previous infection and N-IgG antibodies were more frequent among patients with positive SARS-CoV-2 IGRA results, which suggests that some patients had hybrid immunity induced from previous infection and vaccination. Hybrid immunity has been shown to confer sustained protection against hospital admission or severe disease in infections with the Omicron variant (37), and the mechanism underpinning such protective effect could be the T cell immune response. Remarkably, half of non-vaccinated patients showed a positive SARS-CoV-2 IGRA; of them, one-third had positive TrimericS-IgG antibodies or N-IgG antibodies, which suggests previous infection as the subjacent reason for T cell immunity against SARS-CoV-2. Early cellular immune response developed against acute SARS-CoV-2 infection might have also been involved in the positive T cell responses observed in the rest of the unvaccinated SARS-CoV-2 IGRA-positive patients with negative antibody response. Early cytotoxic CD8+ T cell responses have been observed within 7 days after acute infection and correlated with effective viral clearance and favorable disease outcomes (1, 38). The significantly younger age and lower comorbidity burden of the SARS-CoV-2 IGRA-positive patients might have contributed to a higher frequency of early T cell responses to acute SARS-CoV-2 infection within this group. Finally, a waned antibody response that quickly boosts after reinfection or cross-reactivity with previously infected seasonal coronaviruses (39) could also explain the findings.

Among the SARS-CoV-2 IGRA-positive patients, only one-third showed concomitant non-specific IFN-γ mitogen-stimulated response. The impaired response of the positive control tube (mitogen) to phytohemagglutinin, a non-specific lymphocyte activator, has been correlated with T lymphocyte reduction in the peripheral blood of severely ill patients (32). Accordingly, we observed a significantly lower number of total, T, and CD4 lymphocytes among SARS-CoV-2 IGRA-positive patients with indeterminate mitogen results than in those with positive mitogen. Moreover, the majority of a subgroup of patients with initial negative mitogen results switched to positive after a median of 50 days. Although the interpretation of the positive specific SARS-CoV-2 INF-γ response in the absence of concomitant positive mitogen results is undefined, COVID-19 has been associated with unchanged percentages of positive QuantiFERON-TB despite an increased frequency of low/very low IFN-gamma responses to mitogen (40). This might suggest that SARS-CoV-2 infection would induce a greater impairment in the responses of unstimulated T cells than of viral-stimulated T cells. Interestingly, positive SARS-CoV-2 IGRA patients either with or without mitogen response in our study showed a similar clinical profile when compared with IGRA-negative patients. In both cases, the positive IGRA was associated with younger age, lower comorbidity burden, and lower initial severity. Moreover, mortality was also lower in both groups compared with IGRA-negative patients. According to our results, the performance of the specific SARS-CoV-2 T cell response in patients with severe COVID-19 would not be influenced by the non-specific response of the unstimulated T cells. Remarkably, despite the low frequency of positive mitogen results of the SARS-CoV-2 IGRA, the majority of patients in the cohort showed positive QuantiFERON-TB mitogen. This inconsistency between both mitogen tests may also suggest excessively stringent criteria to define mitogen positivity with the SARS-CoV-2 assays, which could contribute to explain the discrepancy with the results of the specific SARS-CoV-2 IFN-γ responses. Alternatively, the disparity in responses might be explained by the superior sensitivity of the chemiluminescence immunoassay (CLIA) technique used for the quantification of QuantiFERON-TB in comparison to the ELISA method employed in SARS-CoV-2 IGRA (41).

Patients with intermediate/borderline SARS-CoV-2 IGRA results showed several similarities with those with negative SARS-CoV-2 IGRA in demographic characteristics and comorbidity burden, as well as similar lower levels of S-IgG and N-IgG antibodies compared with SARS-CoV-2 IGRA-positive patients. They also exhibited poorer outcomes that were comparable to those of patients with negative SARS-CoV-2 IGRA, including higher frequency of ICU admission and death. Interestingly, the borderline SARS-CoV-2 IGRA group had higher frequency of previous SARS-CoV-2 infection and, although non-significant, of vaccination, and higher T cell and CD4+ T cell counts, but an intermediate frequency of mitogen positivity between SARS-CoV-2 IGRA-positive and SARS-CoV-2 IGRA-negative patients. These findings suggest an impaired T cell response involving not only quantitative but also qualitative abnormalities in the non-positive SARS-CoV-2 IGRA responses. Both the quantity and quality of T cell responses have been linked with disease severity (1, 7, 23, 42). Aging has been associated with altered T cell functionality, including blunted T cell receptor signaling, weakened type I IFN response, or a decline in T cell responsiveness to cytokines (23). This borderline IGRA group may be integrated by patients in whom aging and comorbidity were associated with a predominance of qualitatively over quantitatively impaired T cell immune responses, which would also involve a worse prognosis. This impaired T cell functionality might have induced an imbalanced production of mediators/cytokines with a pro-inflammatory profile, contributing to explain the higher levels of IL-6 and ferritin on admission in this group. Our results suggest that borderline results under 150 mIU/mL should be considered as negative.

The sample size of the study did not allow the evaluation of the SARS-CoV-2 IGRA in unvaccinated patients separately, since they represented a low fraction of the population, and this may be a limitation of the study. We only characterized the test in patients with Omicron and Delta variants, and the performance may not be generalizable to other SARS-CoV-2 variants. Sequential samples were not obtained during hospital stay to analyze changes in the SARS-CoV-2 IGRA results according to disease progression or regression. Further research is needed to confirm the reliability of the SARS-CoV-2-positive results in patients with indeterminate mitogen T cell responses. Strengths are the real-life cohort included in the study, the great number of patients with diverse clinical characteristics and representative of the overall population requiring hospital admission, and the evaluation of the test with different thresholds and types of T cell responses and in more than one confirmed genetic variant.

In conclusion, a specific SARS-CoV-2 IGRA performed during the acute phase of COVID-19 in hospitalized patients revealed a significant proportion of negative results, even among vaccinated individuals. The assay did properly classify patients according to disease severity and prognosis, and testing performance was not influenced by the mitogen results or the Omicron vs. Delta variant. An IFN-γ cut-off > 150 mIU/mL showed a high negative predictive value against mortality. The SARS-CoV-2 IGRA in patients hospitalized for COVID-19 may be a useful tool to assess T cell immunity and adopt targeted therapeutic and preventive measures.

## SUPPLEMENTAL MATERIAL

Supplemental material spectrum.02419-23-s0001.docx Tables S1 to S5, Fig. S1 to S3, and supplemental methods.

ASM does not own the copyrights to Supplemental Material that may be linked to, or accessed through, an article. The authors have granted ASM a non-exclusive, world-wide license to publish the Supplemental Material files. Please contact the corresponding author directly for reuse.
