# Supplementary material for: Integrating SARS-CoV-2-specific interferon-γ release assay testing in the evaluation of patients hospitalized with COVID-19
Source: Microbiol Spectr. 2023 Oct 19;11(6):e02419-23. doi: 10.1128/spectrum.02419-23 (PMC10715100; doi:10.1128/spectrum.02419-23)
Supplement: Supplemental material — Tables S1 to S5, Fig. S1 to S3, and supplemental methods. [file spectrum.02419-23-s0001.docx]

**Supplementary Material**

Assessing the performance of SARS-CoV-2 specific interferon-γ release assay testing in patients hospitalized with COVID-19

**Table of contents**

Supplemental Table S-1…………………………………………………………………………………….2

Supplemental Table S-2……………………………………………………………………………….……5

Supplemental Table S-3…………………………………………………………………….………………6

Supplemental Table S-4…………………………………………………………………………………….7

Supplemental Table S-5…………………………………………………………………………………….9

Supplemental Figure S1…………………………………………………………………………………...10

Supplemental Figure S2…………………………………………………………………………………...11

Supplemental Figure S3…………………………………………………………………………………...11

Supplemental Methods …………………………………………………………………………………...12

**Table S1. SARS-CoV-2 interferon-γ release assay results according to patients´ characteristics at hospital admission with COVID-19.**

| **Characteristic** | **All**  **N (%)** | **Median (Q1-Q3) SARS-CoV-2 IGRA value (mIU/mL)** | **P** | **N (%) Positive SARS-CoV-2 IGRA (>200** **mIU/mL)** | **P** | **N (%) Positive Mitogen-IGRA** | **P** |
| --- | --- | --- | --- | --- | --- | --- | --- |
| N | 248 | 206 (26-636) |  | 125 (50.4) |  | 62 (25.0) |  |
| Sex, Male  Female | 134 (54.0)  114 (46.0) | 185 (24-585)  249 (30-714) | 0.674 | 64 (47.8)  61 (53.5) | 0.376 | 27 (20.1)  35 (30.7) | 0.077 |
| Age, ≤65 y  >65 y | 114 (46.0)  134 (54.0) | 303 (56-1348)  145 (13-430) | <0.001 | 69 (60.5)  56 (41.8) | 0.004 | 31 (27.2)  31 (23.1) | 0.467 |
| Race, White non Hispanic  other | 221 (89.1)  27 (10.9) | 185 (19- 638)  246 (77-578) | 0.350 | 109 (49.3)  16 (59.3) | 0.416 | 56 (25.3)  6 (22.2) | 0.818 |
| Charlson comorbidity index, <4  ≥4 | 119 (48.0)  129 (52.0) | 269 (55-1167)  163 (10-463) | 0.004 | 68 (57.1)  57 (44.2) | 0.043 | 29 (24.4)  33 (25.6) | 0.884 |
| No. of comorbidities*, ≤2  >2 | 156 (62.9)  92 (37.1) | 254 (44-775)  154 (10-542) | 0.043 | 86 (55.1)  39 (42.4) | 0.066 | 37 (23.7)  25 (27.2) | 0.548 |
| Diabetes, Yes  No | 60 (24.2)  188 (75.8) | 163 (14-542)  233 (32-741) | 0.172 | 27 (45.0)  98 (52.1) | 0.375 | 17 (28.3)  45 (23.9) | 0.497 |
| Congestive heart failure, Yes  No | 27 (10.9)  221 (89.1) | 221 (44-844)  203 (27-627) | 0.625 | 14 (51.9)  111 (50.2) | 1.000 | 7 (25.9)  55 (24.9) | 1.000 |
| Coronary artery disease, Yes  No | 30 (12.1)  218 (87.9) | 98 (6-573)  239 (40-637) | 0.136 | 12 (40.0)  113 (51.8) | 0.247 | 6 (20.0)  56 (25.7) | 0.654 |
| Prior stroke, Yes  No | 20 (8.1)  228 (91.9) | 309 (54-761)  198 (22-629) | 0.483 | 12 (60.0)  113 (49.6) | 0.485 | 7 (35.0)  55 (24.1) | 0.288 |
| Peripheral arterial disease, Yes  No | 15 (6.0)  233 (94.0) | 275 (59-519)  198 (22-638) | 0.731 | 9 (60.0)  116 (49.8) | 0.596 | 3 (20.0)  59 (25.3) | 0.768 |
| Pulmonary disease, Yes  No | 53 (21.4)  195 (78.6) | 184 (4-528)  212 (41-704) | 0.258 | 25 (47.2)  100 (51.3) | 0.644 | 12 (22.6)  50 (25.6) | 0.723 |
| Chronic kidney disease, Yes  No | 42 (16.9)  206 (83.1) | 148 (12-274)  247 (34-842) | 0.023 | 16 (38.1)  109 (52.9) | 0.092 | 10 (23.8)  52 (25.2) | 1.000 |
| Malignant neoplasm, Yes  No | 33 (13.3)  215 (86.7) | 212 (30-467)  203 (25-651) | 0.795 | 17 (51.5)  108 (50.2) | 1.000 | 7 (21.2)  55 (25.6) | 0.671 |
| Immunosuppressive condition^£^, Yes  No | 33 (13.3)  215 (86.7) | 272 (42-588)  198 (25-637) | 0.569 | 18 (54.5)  107 (49.8) | 0.709 | 8 (24.2)  54 (25.1) | 1.000 |
| Prior COVID-19 infection, Yes  No | 9 (3.6)  239 (96.4) | 209.(156-339)  203(21-671) | 0.782 | 5 (55.6)  120 (50.2) | 1.000 | 1 (11.1)  61 (25.5) | 0.457 |
| Vaccination status, Vaccinated  Unvaccinated | 181 (73.0)  67 (27.0) | 221 (33-635)  183 (15-644) | 0.649 | 92 (50.8)  33 (49.3) | 0.887 | 51 (28.2)  11 (16.4) | 0.069 |
| **Clinical presentation** |  |  |  |  |  |  |  |
| Days from symptom onset to admission, ≥6  <6 | 134 (54.7)  111 (45.3) | 191 (18-585)  246 (45-722) | 0.353 | 65 (48.5)  59 (53.2) | 0.522 | 41 (30.6)  21 (18.9) | 0.040 |
| SpO2/FiO2 ratio at admission, ≤350  >350 | 138 (55.6)  110 (44.4) | 159 (14-490)  303 (56-1141) | 0.002 | 57 (41.3)  68 (61.8) | 0.001 | 24 (17.4)  38 (34.5) | 0.003 |
| WHO severity score, ≤4  >4 | 143 (57.7)  105 (42.3) | 237 (25-775)  182 (27-528 | 0.458 | 73 (51.0)  52 (49.5) | 0.898 | 44 (30.8)  18 (17.1) | 0.017 |
| X-Ray bilateral lung infiltrates, Yes  No | 18 (47.6)  130 (52.4) | 232 (40-720)  184 (18-611) | 0.441 | 62 (52.5)  63 (48.5) | 0.528 | 21 (17.8)  41 (31.5) | 0.013 |
| **Microbiological data** |  |  |  |  |  |  |  |
| SARS-CoV-2 variant, Omicron  Delta | 142 (57.3)  106 (42.7) | 191 (11-561)  253 (47-916) | 0.095 | 69 (48.6)  56 (52.8) | 0.524 | 39 (27.5)  23 (21.7) | 0.374 |
| Lowest cycle threshold PCR, ≤20  >20 | 84 (33.9)  164 (66.1) | 153 (10-407)  256 (45-713) | 0.037 | 43 (40.2)  82 (58.2) | \|  \| 0.007 \| \| --- \| --- \| | 21 (19.6) 41 (29.1) | 0.104 |
| TrimericS-IgG, Positive  Yes  No | 172 (69.4)  76 (30.6) | 277 (46-871)  85 (4-273) | <0.001 | 99 (57.6)  26 (34.2) | 0.001 | 50 (29.1)  12 (15.8) | 0.027 |
| TrimericS-IgG >264 BAU/mL, Yes  No | 141 (56.9)  107 (43.1) | 293 (49-1087)  145 (6-343) | 0.001 | 82 (58.2)  43 (40.2) | 0.007 | 41 (29.1)  21 (19.6) | 0.104 |
| N-IgG Positive, Yes  No | 34 (13.7)  214 (86.3) | 488 (131-1347)  182 (18-572) | 0.005 | 23 (67.6)  102 (47.7) | 0.041 | 5 (14.7)  57 (26.6) | 0.199 |
| Mitogen-TB-Quantiferon, <0.5  ≥0.50 | 13 (5.9)  207 (94.1) | 92 (4-704)  209 (38-637) | 0.327 | 5 (38.5)  105 (50.7) | 0.569 | 1 (7.7)  52 (25.1) | 0.197 |
| **Laboratory values** |  |  |  |  |  |  |  |
| Interleukin-6, pg/mL (-Inf,15]  (15, Inf] | 68 (27.4)  180 (72.6) | 115 (14-431)  243 (45-856) | 0.057 | 29 (42.6)  96 (53.4) | 0.155 | 12 (17.6)  50 (28.1) | 0.138 |
| C-reactive protein, mg/L, ≤20  >20 | 51 (20.6)  197 (79.4) | 135 (10-925)  209 (42-627) | 0.677 | 25 (49.0)  100 (50.8) | 0.876 | 14 (27.5)  48 (24.4) | 0.717 |
| D-dimer, 𝜇g/mL, ≤0,7  >0,7 | 118 (48.0)  128 (52.0) | 239 (45-1047)  183 (19 -498) | 0.150 | 62 (52.5)  62 (48.4) | 0.527 | 34 (28.8)  28 (21.9) | 0.241 |
| Ferritin, ng/mL, ≤200  >200 | 109 (44.0)  139 (56.0) | 254 (16-739)  180 (43-545) | 0.716 | 60 (55.0)  65 (46.8) | 0.203 | 36 (33.0)  26 (18.7) | 0.012 |
| Total lymphocyte count, cells/𝜇L, ≤700  >700 | 123 (50.6)  120 (49.4) | 161 (18-508  268 (47-943) | 0.058 | 54 (43.9)  69 (57.5) | 0.040 | 20 (16.3)  41 (34.2) | 0.002 |
| B-cell count, cells /𝜇L, ≤65  >65 | 136 (56.2)  106 (43.8) | 169 (16-496)  271 (52-1141) | 0.012 | 62 (45.6)  61 (57.5) | 0.071 | 29 (21.3)  32 (30.2) | 0.136 |
| T-cell count, cells /𝜇L, ≤500  >500 | 140 (57.6)  103 (42.4) | 171 (18-464)  275 (47-1137) | 0.033 | 65 (46.4)  58 (56.3) | 0.153 | 20 (14.3)  41 (39.8) | <0.001 |
| CD4 T-cell count, cells /𝜇L  ≤200  >200 | 98 (40.3  145 (59.7) | 150(12-491)  268 (48-874 | 0.017 | 46 (46.9)  77 (53.1) | 0.363 | 13 (13.3)  48 (33.1) | <0.001 |
| **Antivirals/immunomodulators** |  |  |  |  |  |  |  |
| Remdesivir, Yes  No | 201 (81.0)  47 (19.0) | 185 (29-635)  252 (25-634) | 0.730 | 98 (48.8)  27 (57.4) | 0.332 | 50 (24.9)  12 (25.5) | 1.000 |
| Monoclonal antibodies, Yes  No | 6 (2.4)  242 (97.6) | 6 (1.4-349)  211 (31-637) | 0.205 | 2 (33.3)  123 (50.8) | 0.445 | 1 (16.7)  61 (25.2) | 1.000 |
| Tocilizumab/baricitinib, Yes  No | 163 (65.7)  85 (34.3) | 229 (45-650)  182 (8-618) | 0.259 | 85 (52.1)  40 (47.1) | 0.504 | 38 (23.3)  24 (28.2) | 0.441 |
| **Outcomes** |  |  |  |  |  |  |  |
| Hospital stay, ≤4 days  >4 days | 108 (43.5)  140 (56.5) | 277 (44-1156)  171 (14-518) | 0.020 | 61 (56.5)  64 (45.7) | 0.098 | 33 (30.6)  29 (20.7) | 0.103 |
| In hospital death, Yes  No | 21 (8.5)  227 (91.5) | 10 (0.5-56)  248 (43-722) | <0.001 | 3 (14.3)  122 (53.7) | <0.001 | 0 (0.0)  62 (27.3) | 0.003 |
| ICU admission, Yes  No | 12 (4.8)  236 (95.2) | 54 (22-200)  225 (28-674) | 0.126 | 3 (25.0)  122 (51.7) | 0.083 | 1 (8.3)  61 (25.8) | 0.304 |
| 28-day mortality, Yes  No | 24 (9.7)  224 (90.3) | 20 (2-145)  247 (43-713) | 0.001 | 5 (20.8)  120 (53.6) | 0.002 | 1 (4.2)  61 (27.2) | 0.012 |
| 60-day mortality, Yes  No | 34 (13.7)  214 (86.3) | 52 (5-299)  247 (43-730) | 0.006 | 10 (29.4)  115 (53.7) | 0.010 | 5 (14.7)  57 (26.6) | 0.199 |
| 90-day mortality, Yes  No | 36 (14.5)  212 (85.5) | 49 (4-278)  250 (43-741) | 0.002 | 10 (27.8)  115 (54.2) | 0.004 | 6 (16.7)  56 (26.4) | 0.297 |

Continuous variables are expressed as median (interquartile range). Categorical variables are expressed as number (percentage). * This category included the following underlying medical conditions: diabetes mellitus, chronic cardiac disease, chronic kidney disease, chronic liver disease, chronic neurologic disease, chronic pulmonary disease, malignancies, and immunosuppressive conditions. ^£^ This category included HIV, solid or bone marrow transplant, active hematologic malignancy, receiving immunosuppression, or active chemotherapy. ^&^ A positive result was defined according to the manufacturer as: IFN-γ[mitogen] – IFN-γ[blank] ≥ 400 mIU/mL. Data are presented as no. (%) unless otherwise indicated. IGRA, SARS-CoV-2 interferon gamma release assay; SpO2/FiO2 ratio, oxygen saturation to fraction of inspired oxygen ratio; TrimericS-IgG, immunoglobulin G antibody serum levels against the trimeric spike protein; BAU, Binding Antibody Units; ICU, intensive care unit.

**Table S2. Characteristics of persons admitted to hospital with COVID-19 according to the vaccination status at hospital admission.**

| **Characteristic** | **SARS-CoV-2 unvaccinated** | **SARS-CoV-2 vaccinated** | **All** | ***P* Value** |
| --- | --- | --- | --- | --- |
| No. (%) | 67 (27.0) | 181 (73.0) | 248 |  |
| Sex, male | 32 (47.8) | 102 (56.4) | 134 (54.0) | 0.253 |
| Age, y | 66 (48-79) | 74 (54-83) | 70 (52-83) | 0.045 |
| White, non-Hispanic | 59 (88.1) | 162 (89.5) | 221 (89.1) | 0.819 |
| Charlson comorbidity index | 3 (0-5) | 4 (1-7) | 4 (1-6) | 0.004 |
| Number of comorbidities * | 1 (0-3) | 2 (0-4) | 2 (0-4) | 0.073 |
| Diabetes | 9 (13.4) | 51 (28.2) | 60 (24.2) | 0.019 |
| Congestive heart failure | 5 (7.5) | 22 (12.2) | 27 (10.9) | 0.363 |
| Coronary artery disease | 10 (14.9) | 20 (11.0) | 30 (12.1) | 0.390 |
| Prior stroke | 4 (6.0) | 16 (8.8) | 20 (8.1) | 0.603 |
| Peripheral arterial disease | 2 (3.0) | 13 (7.2) | 15 (6.0) | 0.367 |
| Pulmonary disease | 15 (22.4) | 38 (21.0) | 53 (21.4) | 0.862 |
| Chronic kidney disease | 6 (9.0) | 36 (19.9) | 42 (16.9) | 0.055 |
| Malignant neoplasm | 4 (6.0) | 29 (16.0) | 33 (13.3) | 0.056 |
| Immunosuppressive condition£ | 3 (4.5) | 30 (16.6) | 33 (13.3) | 0.011 |
| Prior COVID-19 infection | 0 (0) | 9 (5.0) | 9 (3.6) | 0.118 |
| **Clinical presentation** |  |  |  |  |
| Days from symptom onset to admission | 7.0 (4.0-8.0) | 5.0 (3.0-7.0) | 5.0 (3.0-7.0) | 0.009 |
| SpO2/FiO2 ratio at admission | 3460 (275-452) | 350 (309-457) | 350 (302-457) | 0.109 |
| WHO severity score | 4.0 (3.1-4.1) | 4.0 (3.1-4.1) | 4.0 (3.1-4.1) | 0.420 |
| X-Ray bilateral lung infiltrates | 41 (61.2) | 77 (42.5) | 118 (47.6) | 0.067 |
| **Microbiological data** |  |  |  |  |
| SARS-CoV-2 variant, Omicron | 39 (58.2) | 103 (56.9) | 142 (57.3) | 0.886 |
| Lowest cycle threshold PCR | 23.8 (19.0-28.8) | 22.1 (17.7-27.3) | 22.3 (18.0-28.5) | 0.186 |
| TrimericS-IgG, BAU/mL | 4.8 (4.8-15) | 1710 (266-5670) | 581 (10-3278) | <0.001 |
| TrimericS-IgG, Positive | 14 (20.9) | 158 (87.3) | 172 (69.4) | <0.001 |
| TrimericS-IgG >264 BAU/mL | 5 (7.5) | 136 (75.1) | 141 (56.9) | <0.001 |
| N-IgG, Positive | 11 (16.4) | 23 (12.7) | 34 (13.7) | 0.533 |
| SARS-CoV-2 IGRA, mIU/mL | 183 (15-644) | 221 (33-635) | 206 (26-636) | 0.649 |
| SARS-CoV-2 IGRA, Positive | 33 (49.3) | 92 (50.8) | 125 (50.4) | 0.887 |
| Mitogen-SARS-CoV-2 IGRA, ^&^ Positive | 11 (16.4) | 51 (28.2) | 62 (25.0) | 0.069 |
| **First available laboratory values** |  |  |  |  |
| Interleukin-6, pg/mL | 45 (21-140) | 42 (12-139) | 42 (12-139) | 0.531 |
| C-reactive protein, mg/L | 48 (26-92) | 52 (23-98) | 50 (24-97) | 0.839 |
| D-dimer, 𝜇g/mL | 0.7 (0.5-1.4) | 0.8 (0.4-1.4) | 0.7 (0.4-1.4) | 0.833 |
| Ferritin, ng/mL | 296 (150-624) | 221 (99-487) | 238 (109-511) | 0.051 |
| Peripheral lymphocyte count, cells/𝜇L | 659 (404-1021) | 699 (487-999) | 694 (461-1013) | 0.428 |
| B-cell count, cells /𝜇L | 71 (38-105) | 54 (25-117) | 60 (28-113) | 0.143 |
| T-cell count, cells /𝜇L | 366 (255-598) | 455 (304-703) | 435 (281-660) | 0.094 |
| CD4 T-cell count, cells /𝜇L | 214 (127-371) | 249 (154-376) | 240 (146-376) | 0.384 |
| CD4/CD8 lymphocyte ratio | 1.9 (1.1-2.6) | 1.5 (1.0-2.3) | 1.6 (1.0-2.4) | 0.107 |
| **Antivirals/immunomodulators** |  |  |  |  |
| Remdesivir | 57 (85.1) | 144 (79.6) | 201 (81.0) | 0.367 |
| Monoclonal antibodies | 1 (1.5) | 5 (2.8) | 6 (2.4) | 1.000 |
| Tocilizumab or baricitinib | 45 (67.2) | 118 (65.2) | 163 (65.7) | 0.880 |
| **Outcomes** |  |  |  |  |
| Hospital stay, days | 6.0 (4.0-8.0) | 5.0 (3.0-8.0) | 5.0 (3.0-8.0) | 0.478 |
| ICU admission | 4 (6.0) | 8 (4.4) | 12 (4.8) | 0.739 |
| 28-day mortality | 7 (10.4) | 17 (9.4) | 24 (9.7) | 0.811 |
| 60-day mortality | 7 (10.4) | 27 (14.9) | 34 (13.7) | 0.413 |
| 90-day mortality | 8 (11.9) | 28 (15.5) | 36 (14.5) | 0.548 |

Continuous variables are expressed as median (interquartile range). Categorical variables are expressed as number (percentage). *This category included the following underlying medical conditions: diabetes mellitus, chronic cardiac disease, chronic kidney disease, chronic liver disease, chronic neurologic disease, chronic pulmonary disease, malignancies, and immunosuppressive conditions. ^£^ This category included HIV, solid or bone marrow transplant, active hematologic malignancy, receiving immunosuppression, or active chemotherapy. ^&^ A positive result was defined according to the manufacturer as: IFN-γ[mitogen] – IFN-γ[blank] ≥ 400 mIU/mL. Data are presented as no. (%) unless otherwise indicated. IGRA, SARS-CoV-2 interferon gamma release assay; SpO2/FiO2 ratio, oxygen saturation to fraction of inspired oxygen ratio; TrimericS-IgG, immunoglobulin G antibody serum levels against the trimeric spike protein; BAU, Binding Antibody Units; ICU, intensive care unit.

**Table S3. Characteristics of persons admitted to hospital with COVID-19 according to the SARS-CoV-2 variant at hospital admission.**

| **Characteristic** | **Delta variant** | **Omicron variant** | **All** | ***P* Value** |
| --- | --- | --- | --- | --- |
| No. (%) | 106 (42.7) | 142 (57.3) | 248 |  |
| Sex, male | 61 (57.5) | 73 (51.4) | 134 (54.0) | 0.369 |
| Age, y | 61 (50-76) | 77 (57-85) | 70 (52- 83) | <0.001 |
| White, non Hispanic | 91 (85.8) | 130 (91.5) | 221 (89.1) | 0.216 |
| Charlson comorbidity index | 2 (1-5) | 5 (2-7) | 4 (1- 6) | <0.001 |
| Number of comorbidities^*^ | 1 (0-3) | 2 (0.2-4) | 2 (0-4) | <0.001 |
| Diabetes | 24 (22.6) | 36 (25.4) | 60 (24.2) | 0.655 |
| Congestive heart failure | 7 (6.6) | 20 (14.1) | 27 (10.9) | 0.067 |
| Coronary artery disease | 9 (8.5) | 21 (14.8) | 30 (12.1) | 0.169 |
| Prior stroke | 5 (4.7) | 15 (10.6) | 20 (8.1) | 0.105 |
| Peripheral arterial disease | 4 (3.8) | 11 (7.7) | 15 (6.0) | 0.282 |
| Pulmonary disease | 16 (15.1) | 37 (26.1) | 53 (21.4) | 0.042 |
| Chronic kidney disease | 14 (13.2) | 28 (19.7) | 42 (16.9) | 0.231 |
| Malignant neoplasm | 10 (9.4) | 23 (16.2) | 33 (13.3) | 0.134 |
| Immunosuppressive condition^£^ | 8 (7.5) | 25 (17.6) | 33 (13.3) | 0.023 |
| Prior COVID-19 infection | 3 (2.8) | 6 (4.2) | 9 (3.6) | 0.736 |
| **Vaccination status** |  |  |  |  |
| Unvaccinated | 28 (26.4) | 39 (27.5) | 67 (27.0) | 0.886 |
| Days since last vaccine dose | 129 (69-180) | 94 (67-176) | 110 (67-178) | 0.239 |
| **Clinical presentation** |  |  |  |  |
| Days from symptom onset to admission | 6 (4-7) | 4 (2-7) | 5 (3-7) | 0.004 |
| SpO2/FiO2 ratio at admission | 346 (306-457) | 350 (300-452) | 350 (302-457) | 0.984 |
| WHO severity score | 4.0 (3.1 to 4.1) | 3.9 (3.0 to 4.0) | 4.0 (3.1 to 4.1) | 0.287 |
| Bilateral lung infiltrates on X Ray | 58 (54.7) | 60 (42.3) | 118 (47.6) | 0.098 |
| **Microbiological data** |  |  |  |  |
| SARS-CoV-2 variant, Omicron | 0 | 142 | 142 |  |
| Lowest cycle threshold PCR detected | 24 (19-30) | 21 (18-28) | 22 (18-29) | 0.040 |
| Sampling time from admission, days | 1 (1-2) | 1 (1 -1) | 1 (1-1) | 0.050 |
| TrimericS-IgG, BAU/mL | 488 (18-4748) | 745 (8-2308) | 581 (10-3278) | 0.455 |
| TrimericS-IgG, Positive | 78 (73.6) | 94 (66.2) | 172 (69.4) | 0.265 |
| TrimericS-IgG >264 BAU/mL |  |  |  |  |
| N-IgG, Positive | 17 (16.0) | 17 (12.0) | 34 (13.7) | 0.359 |
| SARS-CoV-2 IGRA, mIU/mL | 253 (47-916) | 191 (11-561) | 206 (26-636) | 0.095 |
| SARS-CoV-2 IGRA, Positive | 56 (52.8) | 69 (48.6) | 125 (50.4) | 0.524 |
| Mitogen-SARS-CoV-2 IGRA, & Positive | 23 (21.7) | 39 (27.5) | 62 (25.0) | 0.374 |
| **First available laboratory values** |  |  |  |  |
| Interleukin-6, pg/mL | 49 (18-133) | 34 (8-140) | 42 (12-139) | 0.133 |
| C-reactive protein, mg/L | 61 (29-99) | 39 (20-89) | 50 (24-97) | 0.019 |
| D-dimer, 𝜇g/mL | 0.6 (0.4 to 1.0) | 0.8 (0.5 to 1.9) | 0.7 (0.4 to 1.4) | 0.016 |
| Ferritin, ng/mL | 378 (163-546) | 172 (84- 462) | 238 (109-511) | <0.001 |
| Total lymphocyte count, cells/𝜇L | 798 (456-1066.0) | 673 (461-973.5) | 694 (461-1013) | 0.213 |
| B-cell count, cells /𝜇L | 59 (32-109) | 60 (24-114) | 60 (28-113) | 0.580 |
| T-cell count, cells /𝜇L | 452 (280-738) | 430 (285-626) | 435 (281-660) | 0.446 |
| CD4 T-cell count, cells /𝜇L | 264 (140-414.0) | 225 (150-370.8) | 240 (146-376) | 0.702 |
| CD4/CD8 lymphocyte ratio | 1.4 (0.9 to 2.3) | 1.8 (1.2 to 2.4) | 1.6 (1.0 to 2.4) | 0.042 |
| **Antivirals/immunomodulators** |  |  |  |  |
| Remdesivir | 86 (81.1) | 115 (81.0) | 201 (81.0) | 1.000 |
| Monoclonal antibodies | 1 (0.9) | 5 (3.5) | 6 (2.4) | 0.243 |
| Tocilizumab or baricitinib | 84 (79.2) | 79 (55.6) | 163 (65.7) | <0.001 |
| **Outcomes** |  |  |  |  |
| Hospital stay, days | 4.5 (3.0 to 7.0) | 6.0 (4.0 to 8.0) | 5.0 (3.0 to 8.0) | 0.197 |
| ICU admission | 8 (7.5) | 4 (2.8) | 12 (4.8) | 0.132 |
| 28-day mortality | 9 (8.5) | 15 (10.6) | 24 (9.7) | 0.667 |
| 60-day mortality | 11 (10.4) | 23 (16.2) | 34 (13.7) | 0.198 |
| 90-day mortality | 11 (10.4) | 25 (17.6) | 36 (14.5) | 0.145 |

Continuous variables are expressed as median (interquartile range). Categorical variables are expressed as number (percentage). *This category included the following underlying medical conditions: diabetes mellitus, chronic cardiac disease, chronic kidney disease, chronic liver disease, chronic neurologic disease, chronic pulmonary disease, malignancies, and immunosuppressive conditions. ^£^ This category included HIV, solid or bone marrow transplant, active hematologic malignancy, receiving immunosuppression, or active chemotherapy. ^&^ A positive result was defined according to the manufacturer as: IFN-γ[mitogen] – IFN-γ[blank] ≥ 400 mIU/mL. Data are presented as no. (%) unless otherwise indicated. IGRA, SARS-CoV-2 interferon gamma release assay; SpO2/FiO2 ratio, oxygen saturation to fraction of inspired oxygen ratio; TrimericS-IgG, immunoglobulin G antibody serum levels against the trimeric spike protein; BAU, Binding Antibody Units; ICU, intensive care unit.

**Table S4. Univariate analysis of 28-day mortality in persons admitted-hospital with COVID-19.**

| **Characteristic** | **28-day death** | **Non-28-day death** | **All** | **P Value** |
| --- | --- | --- | --- | --- |
| n (%) | 24 (9.7) | 224 (90.3) | 248 |  |
| Sex, male | 11 (45.8) | 123 (54.9) | 134 | 0.519 |
| Age, y | 87 (81-89) | 66 (51-81) | 70 (52-83) | <0.001 |
| Charlson comorbidity index | 7 (5-9) | 3 (1-6) | 4 (1-6) | <0.001 |
| Number of comorbidities,* | 4 (2-5) | 1 (0-3) | 2 (0-4) | <0.001 |
| Diabetes | 8 (33.3) | 52 (23.2) | 60 (24.2) | 0.315 |
| Congestive heart failure | 4 (16.7) | 23 (10.3) | 27 (10.9) | 0.310 |
| Coronary artery disease | 9 (37.5) | 21 (9.4) | 30 (12.1) | 0.001 |
| Prior stroke | 5 (20.8) | 15 (6.7) | 20 (8.1) | 0.032 |
| Peripheral arterial disease | 2 (8.3) | 13 (5.8) | 15 (6.0) | 0.645 |
| Pulmonary disease | 5 (20.8) | 48 (21.4) | 53 (21.4) | 1.000 |
| Chronic kidney disease | 9 (37.5) | 33 (14.7) | 42 (16.9) | 0.009 |
| Malignant neoplasm | 7 (29.2) | 26 (11.6) | 33 (13.3) | 0.025 |
| Immunosuppressive condition£ | 4 (16.7) | 29 (12.9) | 33 (13.3) | 0.538 |
| Prior COVID-19 infection | 0 | 9 (4.0) | 9 (3.6) | 1.000 |
| **Vaccination status** |  |  |  |  |
| Days since last vaccine dose | 118 (85-61) | 105 (66-179) | 110 (67-78) | 0.475 |
| Unvaccinated | 7 (29.2) | 60 (26.8) | 67 (27.0) | 0.811 |
| Full vaccinated | 17 (70.8) | 164 (73.2) | 181 (73.0) |  |
| **Clinical presentation** |  |  |  |  |
| Days from symptom onset-admission | 4 (3-8) | 5 (3-7) | 5 (3-7) | 0.563 |
| SpO2/FiO2 ratio at admission | 2.7 (2.3-3.5) | 3.5 (3.1-4.6) | 3.5 (3.0-4.6) | 0.563 |
| WHO severity score >4 | 5 (20.8) | 13 (5.8) | 18 (7.3) | 0.020 |
| X-Ray bilateral lung infiltrates | 12 (50.0) | 106 (47.3) | 118 (47.6) | 0.615 |
| **Microbiological data** |  |  |  |  |
| SARS-CoV-2 variant, Omicron | 15 (62.5) | 127 (56.7) | 142 (57.3) | 0.667 |
| Lowest cycle threshold PCR | 22 (17-26) | 23 (18-29) | 22 (18-29) | 0.319 |
| TrimericS-IgG, BAU/mL | 978 (5-5673) | 580 (14-2808) | 581 (10-3278) | 0.994 |
| TrimericS-IgG, Positive | 14 (58.3) | 158 (70.5) | 172 (69.4) | 0.246 |
| TrimericS-IgG >264 BAU/mL | 13 (54.2) | 128 (57.1) | 141 (56.9) | 0.830 |
| N-IgG, Positive | 4 (16.7) | 30 (13.4) | 34 (13.7) | 0.753 |
| SARS-CoV-2 IGRA,mIU/mL | 20 (2-145) | 247 (43-713) | 206 (26-636) | 0.001 |
| SARS-CoV-2 IGRA, Positive | 5 (20.8) | 120 (53.6) | 125 (50.4) | 0.002 |
| Mitogen-SARS-CoV-2 IGRA,& Positive | 1 (4.2) | 61 (27.2) | 62 (25.0) | 0.012 |
| Mitogen-QuantiferonTB, medi(IQR) | 7.0 (1.1-9.8) | 9.7 (3.4-10.0) | 9.7 (3.2-10.0) | 0.143 |
| **Laboratory values** |  |  |  |  |
| Interleukin-6, pg/mL | 140 (55-450) | 38 (11-123) | 42 (12-139) | <0.001 |
| C-reactive protein, mg/L | 75 (45-159) | 48 (22-93) | 50 (24-97) | 0.022 |
| D-dimer, 𝜇g/mL | 1.1 (0.7-3.6) | 0.7 (0.4-1.3) | 0.7 (0.4-1.4) | 0.001 |
| Ferritin, ng/mL | 620 (190-910) | 229 (101-489) | 238 (109-511) | 0.003 |
| Peripheral lymphocyte count, cells/𝜇L | 681 (429-962) | 697 (462-1024) | 694 (461-1013) | 0.532 |
| B-cell count, cells/𝜇L | 29 (20-59) | 61 (31-117) | 60 (28-113) | 0.015 |
| T-cell count, cells/𝜇L | 455 (269-574) | 431 (281-684) | 435 (281-660) | 0.686 |
| CD4 T-cell count, cells/𝜇L | 257 (160-382) | 239 (145-376) | 240 (146-376) | 0.903 |
| CD4/CD8 lymphocyte ratio | 1.6 (1.1-2.1) | 1.6 (1.0-2.4) | 1.6 (1.0-2.4) | 0.799 |
| **Antivirals/immunomodulators** |  |  |  |  |
| Remdesivir | 15 (62.5) | 186 (83.0) | 201 (81.0) | 0.025 |
| Monoclonal antibodies | 0 (0.0) | 6 (2.7) | 6 (2.4) | 1.000 |
| Tocilizumab or baricitinib | 12 (50.0) | 151 (67.4) | 163 (65.7) | 0.113 |

Continuous variables are expressed as median (interquartile range). Categorical variables are expressed as number (percentage). *This category included the following underlying medical conditions: diabetes mellitus, chronic cardiac disease, chronic kidney disease, chronic liver disease, chronic neurologic disease, chronic pulmonary disease, malignancies, and immunosuppressive conditions. ^£^ This category included HIV, solid or bone marrow transplant, active hematologic malignancy, receiving immunosuppression, or active chemotherapy. ^&^ A positive result was defined according to the manufacturer as: IFN-γ[mitogen] – IFN-γ[blank] ≥ 400 mIU/mL. Data are presented as no. (%) unless otherwise indicated. IGRA, SARS-CoV-2 interferon gamma release assay; SpO2/FiO2 ratio, oxygen saturation to fraction of inspired oxygen ratio; TrimericS-IgG, immunoglobulin G antibody serum levels against the trimeric spike protein; BAU, Binding Antibody Units; ICU, intensive care unit.

**Table S5. Characteristics of persons admitted to hospital with COVID-19 according to the presence of SARS-CoV-2 cellular response at hospital admission**

| **Characteristic** | **Negative/ borderline**  **SARS-CoV-2 IGRA (<200 mIU/mL)** | **Positive**  **SARS-CoV-2 IGRA (>200 mIU/mL)** | **All** | ***P* Value** |
| --- | --- | --- | --- | --- |
| No, % | 123 (49.6) | 125 (50.4) | 248 |  |
| Sex, male | 70 (56.9) | 64 (51.2) | 134 (54.0) | 0.376 |
| Age, y | 76 (59-85) | 62 (49-79) | 70 (52-83) | <0.001 |
| White, non-Hispanic | 112 (91.1) | 109 (87.2) | 221 (89.1) | 0.416 |
| Charlson comorbidity index | 4 (2-7) | 3 (1-6) | 4 (1-6) | 0.024 |
| Number of comorbidities* | 2 (1-4) | 1 (0-3) | 2 (0-4) | 0.024 |
| Diabetes | 33 (26.8) | 27 (21.6) | 60 (24.2) | 0.375 |
| Congestive heart failure | 18 (14.6) | 12 (9.6) | 30 (12.1) | 0.247 |
| Coronary artery disease | 13 (10.6) | 14 (11.2) | 27 (10.9) | 1.000 |
| Prior stroke | 8 (6.5) | 12 (9.6) | 20 (8.1) | 0.485 |
| Peripheral arterial disease | 6 (4.9) | 9 (7.2) | 15 (6.0) | 0.596 |
| Pulmonary disease | 28 (22.8) | 25 (20.0) | 53 (21.4) | 0.644 |
| Chronic kidney disease | 26 (21.1) | 16 (12.8) | 42 (16.9) | 0.092 |
| Malignant neoplasm | 16 (13.0) | 17 (13.6) | 33 (13.3) | 1.000 |
| Immunosuppressive condition£ | 15 (12.2) | 18 (14.4) | 33 (13.3) | 0.709 |
| Prior COVID-19 infection | 4 (3.3) | 5 (4.0) | 9 (3.6) | 1.000 |
| Vaccination status |  |  |  |  |
| Unvaccinated | 34 (27.6) | 33 (26.4) | 67 (27.0) | 0.887 |
| Vaccinated# | 89 (72.4) | 92 (73.6) | 181 (73.0) |  |
| Days since last vaccine dose | 12 (73-184) | 95 (59-175) | 110 (67-178) | 0.117 |
| Clinical presentation |  |  |  |  |
| Days from symptom onset to admission | 5 (3-7) | 6 (3-8) | 5 (3- 7) | 0.377 |
| SpO2/FiO2 ratio at admission (*100) | 3.4 (2.8-4.1) | 3.5 (3.4-4.6) | 3.5 (3.0-4.6) | <0.001 |
| WHO severity score  WHO severity score >4 | 4.0 (3.1-4.1)  14 (11.4) | 4.0 (3.0-4.0)  4 (3.2) | 4.0 (3.1-4.1)  18 (7.2) | 0.187  0.03 |
| X-Ray bilateral lung infiltrates | 56 (45.5) | 62 (49.6) | 118 (47.6) | 0.298 |
| Microbiological data |  |  |  |  |
| SARS-CoV-2 variant, Omicron | 73 (59.3) | 69 (55.2) | 142 (57.3) | 0.524 |
| Lowest cycle threshold PCR | 22 (17-29) | 23 (19-28) | 22 (18-29) | 0.130 |
| TrimericS-IgG, BAU/mL | 173 (4.8-2535) | 1090 (81-3650) | 581 (10-3278) | 0.015 |
| TrimericS-IgG, Positive | 73 (59.3) | 99 (79.2) | 172 (69.4) | 0.001 |
| TrimericS-IgG >264 BAU/mL | 59 (48.0) | 82 (65.6) | 141 (56.9) | 0.007 |
| N-IgG, Positive | 11 (8.9) | 23 (18.4) | 34 (13.7) | 0.041 |
| SARS-CoV-2 IGRA, mIU/mL | 22 (0.5-80) | 635 (337-1752) | 206 (26-636) | <0.001 |
| Mitogen-SARS-CoV-2 IGRA& Positive | 18 (14.6) | 44 (35.2) | 62 (25.0) | <0.001 |
| Mitogen-QuantiferonTB | 8.6 (2.4-10.0) | 9.8 (3.4-9.9) | 9.7 (3.2-10.0) | 0.827 |
| QuantiferonTB, Positive | 4 (3.5) | 4 (3.6) | 8 (3.6) | 0.902 |
| Outcomes |  |  |  |  |
| Hospital stay, days | 6 (4-9) | 5 (3-7) | 5 (3-8) | 0.092 |
| ICU admission | 9 (7.3) | 3 (2.4) | 12 (4.8) | 0.083 |
| 28-day mortality | 19 (15.4) | 5 (4.0) | 24 (9.7) | 0.002 |
| 60-day mortality | 24 (19.5) | 10 (8.0) | 34 (13.7) | 0.010 |
| 90-day mortality | 6 (21.1) | 10 (8.0) | 36 (14.5 | 0.004 |

Continuous variables are expressed as median (interquartile range). Categorical variables are expressed as number (percentage). *This category included the following underlying medical conditions: diabetes mellitus, chronic cardiac disease, chronic kidney disease, chronic liver disease, chronic neurologic disease, chronic pulmonary disease, malignancies, and immunosuppressive conditions. ^£^This category included HIV, solid or bone marrow transplant, active hematologic malignancy, receiving immunosuppression, or active chemotherapy. ^#^Of 181 vaccinated patients, 103 (57%) were fully vaccinated and 78 (43%) had received a booster dose. ^&^A positive result was defined according to the manufacturer as: IFN-γ[mitogen] – IFN-γ[blank] ≥ 400 mIU/mL. Data are presented as no. (%) unless otherwise indicated. IGRA, SARS-CoV-2 interferon gamma release assay; SpO2/FiO2 ratio, oxygen saturation to fraction of inspired oxygen ratio; TrimericS-IgG, immunoglobulin G antibody serum levels against the trimeric spike protein; BAU, Binding Antibody Units; ICU, intensive care unit.

**Fig. S1. Correlation of interferon-ɣ levels with inflammatory biomarkers and IgG anti-spike antibodies in persons hospitalized with COVID-19.**


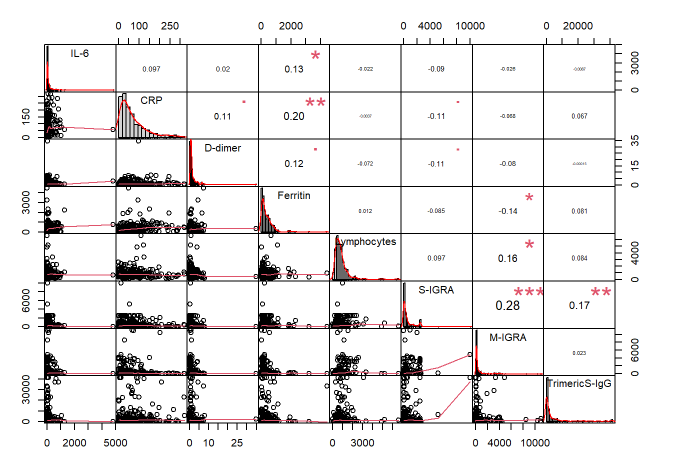


IL-6, Interleukin-6; CRP, C-reactive protein; Lymphocytes, total peripheral lymphocyte count; S-IGRA, SARS-CoV-2 IGRA; M-IGRA, non-specific (mitogen) IGRA response; TrimericS-IgG, immunoglobulin G antibody serum levels against the trimeric spike protein; *p<0.01, **p <0.001 ***p<0.0001.

**Fig. S2. Receiver operating characteristic curve to assess the performance of SARS-CoV-2 interferon-γ assay to predict 28-day mortality.**


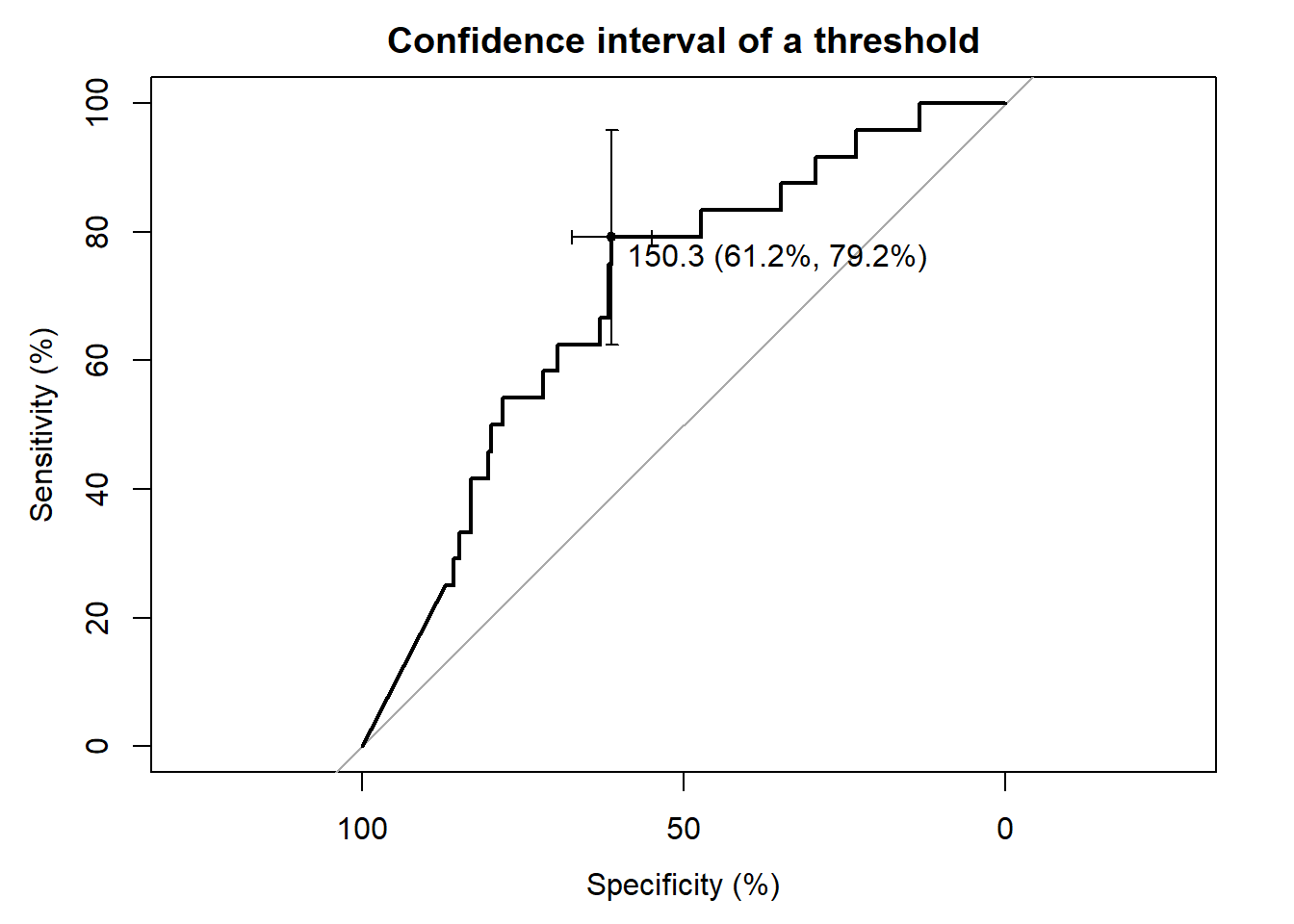


**Fig. S3 Kaplan Meier estimated survival at 28 days curves according to the variables of interest.**

**
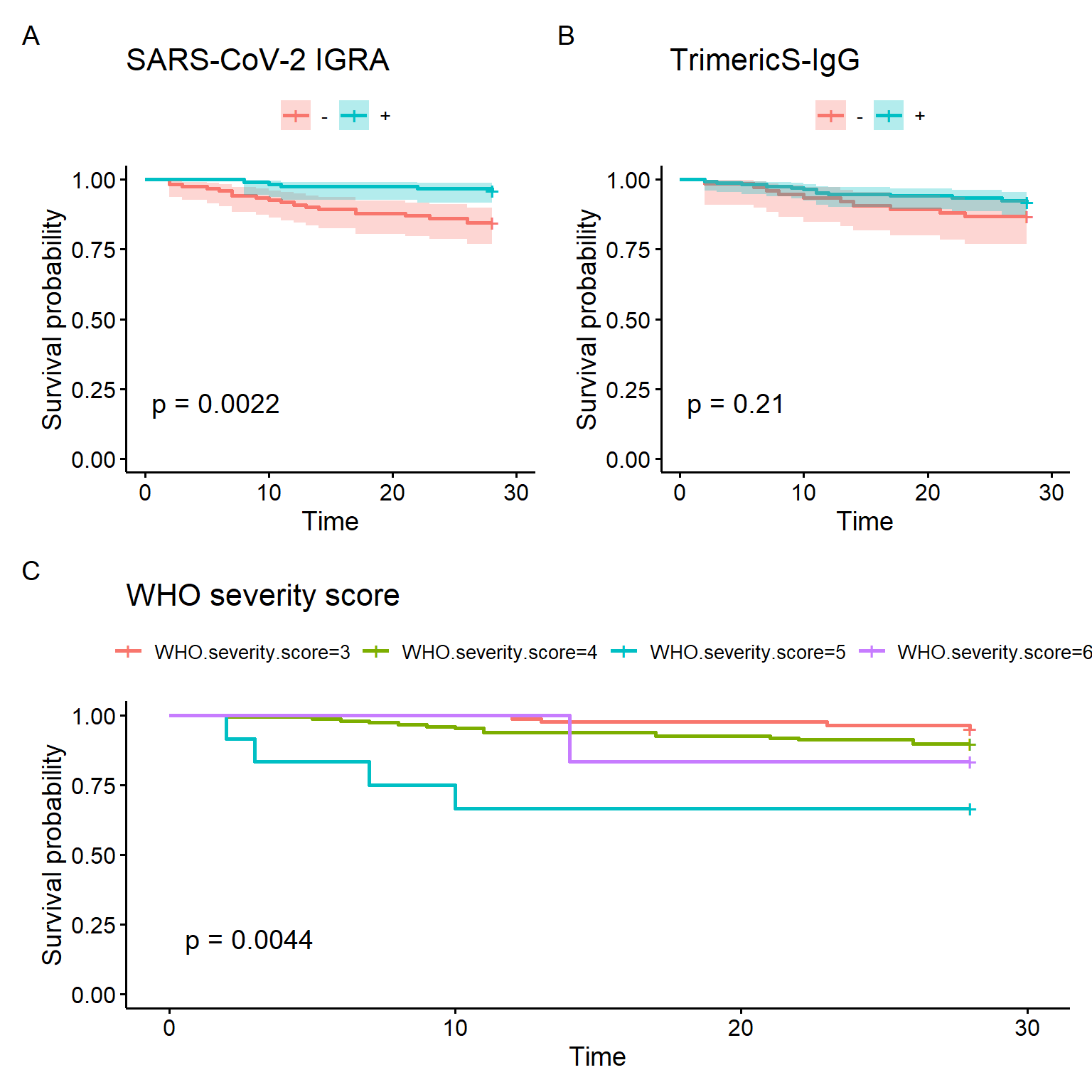
**

**Methods**

**Novaplex SARS-CoV-2 Variants VII Assay (RUO) - Specifications and Methodology**

The NovaplexTM assay targets specific nucleic acid sequences associated with critical Spike protein mutations (E484A, N501Y, and HV69/70 deletion) and the RdRP gene of SARS-CoV-2.

Multiplex Real-time PCR:

The assay employs a multiplex real-time PCR approach, allowing simultaneous amplification of multiple target sequences in a single reaction. This streamlined process not only saves time and resources but also ensures the sensitivity and specificity of the detection, reducing the risk of false-negative or false-positive results.

To achieve superior accuracy and efficiency in target amplification and detection, Seegene's proprietary DPO™ (Dual Priming Oligonucleotide) and TOCE™ (Tagging Oligonucleotide Cleavage and Extension) technologies are employed. DPO™ technology enhances the specificity and sensitivity of primer binding, reducing non-specific amplification, while TOCE™ technology enables robust and accurate target detection.

Please note that the primer sequences used in this assay are proprietary and considered confidential information of Seegene.

Thermal Conditions

The PCR amplification is performed using the following thermal cycling conditions:


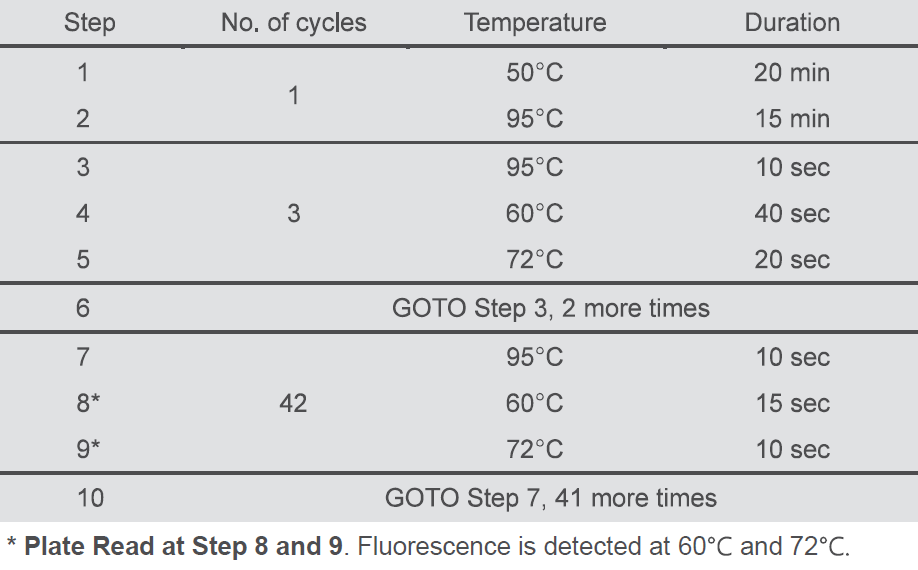


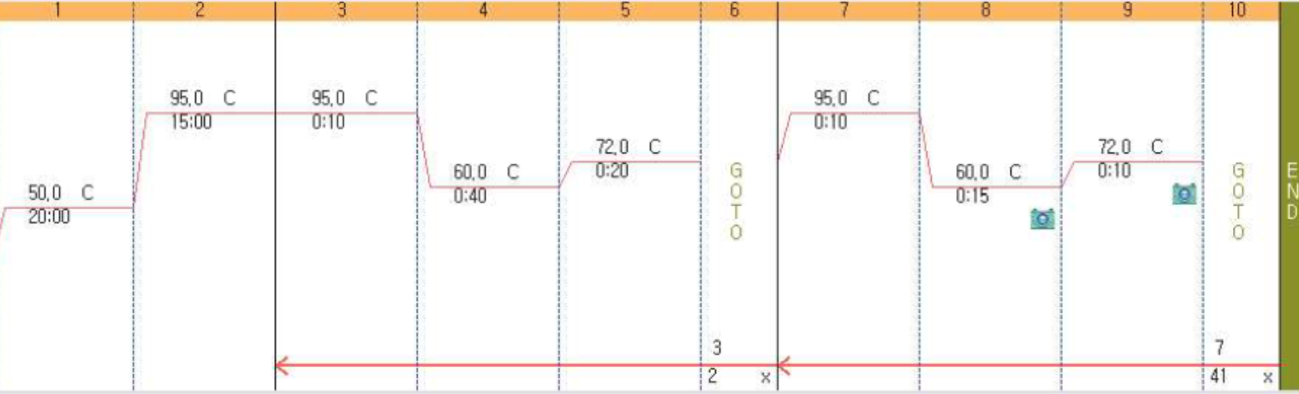
 Fig. M1. Protocol Editor Novaplex SARS-CoV-2 Variants VII Assay.
